# Impact of Gba2 on neuronopathic Gaucher’s disease and α-synuclein accumulation in medaka (*Oryzias latipes*)

**DOI:** 10.1186/s13041-021-00790-x

**Published:** 2021-05-10

**Authors:** Etsuro Nakanishi, Norihito Uemura, Hisako Akiyama, Masato Kinoshita, Sawamura Masanori, Yosuke Taruno, Hodaka Yamakado, Shu-ichi Matsuzawa, Shunichi Takeda, Yoshio Hirabayashi, Ryosuke Takahashi

**Affiliations:** 1 Department of Neurology, Kyoto University Graduate School of Medicine, Kyoto, 606-8507 Japan; 2 Laboratory for Neural Cell Dynamics, RIKEN Center for Brain Science, Saitama, 351-0198 Japan; 3 Division of Applied Bioscience, Kyoto University Graduate School of Agriculture, Kyoto, 606-8502 Japan; 4 Department of Radiation Genetics, Kyoto University Graduate School of Medicine, Kyoto, 606-8507 Japan; 5 Cellular Informatics Laboratory, RIKEN, Saitama, 351-0198 Japan; 6 Present Address: Department of Pathology and Laboratory Medicine, Institute On Aging and Center for Neurodegenerative Disease Research, University of Pennsylvania School of Medicine, Philadelphia, PA 19104-2676 USA

**Keywords:** GBA1, GBA2, Gaucher’s disease, Parkinson’s disease, α-Synuclein, Glucocerebrosidase, Sphingolipids

## Abstract

Homozygous mutations in the lysosomal glucocerebrosidase gene, *GBA1*, cause Gaucher’s disease (GD), while heterozygous mutations in *GBA1* are a strong risk factor for Parkinson’s disease (PD), whose pathological hallmark is intraneuronal α-synuclein (asyn) aggregates. We previously reported that *gba1* knockout (KO) medaka exhibited glucosylceramide accumulation and neuronopathic GD phenotypes, including short lifespan, the dopaminergic and noradrenergic neuronal cell loss, microglial activation, and swimming abnormality, with asyn accumulation in the brains. A recent study reported that deletion of GBA2, non-lysosomal glucocerebrosidase, in a non-neuronopathic GD mouse model rescued its phenotypes. In the present study, we generated *gba2* KO medaka and examined the effect of Gba2 deletion on the phenotypes of *gba1* KO medaka. The Gba2 deletion in *gba1* KO medaka resulted in the exacerbation of glucosylceramide accumulation and no improvement in neuronopathic GD pathological changes, asyn accumulation, or swimming abnormalities. Meanwhile, though *gba2* KO medaka did not show any apparent phenotypes, biochemical analysis revealed asyn accumulation in the brains. *gba2* KO medaka showed a trend towards an increase in sphingolipids in the brains, which is one of the possible causes of asyn accumulation. In conclusion, this study demonstrated that the deletion of Gba2 does not rescue the pathological changes or behavioral abnormalities of *gba1* KO medaka, and GBA2 represents a novel factor affecting asyn accumulation in the brains.

**Supplementary Information:**

The online version contains supplementary material available at 10.1186/s13041-021-00790-x.

## Introduction

Homozygous mutations in *GBA1* are responsible for Gaucher’s disease (GD), the most common lysosomal storage disorder. GBA1 gene encodes the lysosomal glucocerbrosidase (GCase), and mutations in the GBA1 gene lead to a decrease in lysosomal GCase activity. The marked decrease in GCase activity causes the accumulation of the GCase substrate, glucosylceramide (GlcCer) and glucosylsphigosine (GlcSph), which is thought to cause GD [1].

GD is divided into three subtypes based on clinical manifestations: a non-neuronopathic form (type 1: GD1), an acute neuronopathic form (type 2: GD2), and a chronic neuronopathic form (type 3: GD3). GD2 and GD3 are together referred to as neuronopathic GD (nGD) [2]. Recent genetic studies identified the heterozygous mutations in *GBA1* as a strong risk for sporadic Parkinson’s disease (PD) and dementia with Lewy bodies, whose pathological hallmark is the disease-specific inclusion bodies, composed of α-synuclein (asyn) called Lewy bodies [3–5]. Not only heterozygous mutations of GBA1 gene carriers but also GD1 patients are at an increased risk for developing PD [6, 7]. Pathological analysis revealed the presence of Lewy bodies in the brains of both GD1 patients and *GBA1* heterozygotes with PD (*GBA1*-PD), suggesting that *GBA1* mutations contribute to asyn aggregation [8–12]. Accumulating evidence suggests that the perturbation of sphingolipid metabolism and disruption of the autophagic-lysosomal pathway (ALP) play a key role in *GBA1* mutations leading to asyn aggregation [13–20]. However, the underlying mechanisms by which *GBA1* mutations lead to both GD and PD have yet to be established.

Besides GBA1, mammalian GBA2 (non-lysosomal GCase), GBA3 (cytosolic GCase) and lactase-phlorizin hydrolase (LPH) have GCase activity, which hydrolyzes GlcCer into ceramide and glucose. The physiological functions of GBA2 have been investigated in several studies, but there are very few reports on GBA3. LPH is exclusively present in the plasma membrane of the small intestine and may be involved in the digestion of dietary GlcCer [21]. Homozygous loss of function *GBA2* mutations are responsible for hereditary spastic paraplegia (HSP; SPG46), autosomal-recessive cerebellar ataxia (ARCA) and Marinesco-Sjögren-like syndrome in humans [22–27]. A previous study has reported that the deletion of GBA2 resulted in spermatogenesis abnormality but not in CNS abnormality in mice [28]. However, a recent study showed that *Gba2* knockout (KO) mice exhibited locomotor dysfunction despite a high phenotypic variance [29]. Morpholino antisense oligonucleotide-mediated knockdown of *gba2* leads to abnormal motoneuron outgrowth and motility defect in zebrafish [25].

Interestingly, a previous study reported that the deletion of GBA2 rescued the visceral manifestations, such as hepatosplenomegaly, cytopenia and osteopenia, in a GD1 mouse model through a reduction in sphingosine [30]. In addition, the deletion of GBA2 also rescues the CNS phenotypes, such as neuronal cell loss, motor coordination and lifespan, in the Niemann-Pick Type C (NPC) mouse model, which shows a decreased GBA1 activity in the brain [31]. These findings raise the possibility that GBA2 works as a novel factor affecting the CNS pathology in *GBA1*-related disorders, including nGD and PD.

GBA1 and GBA2 are widely conserved from *Caenorhabditis elegans* to humans, as well as in medaka, mice and rats. We previously reported that *gba1* KO medaka survived long enough to perform pathological analysis of the disease progression, in contrast to the perinatal death observed in *Gba1* KO mice [32, 33]. *gba1* KO medaka develop swimming abnormalities at 2 months post-fertilization (mpf) and start to die from 3 mpf. Medaka have endogenous asyn, enabling the observation of the effects of genetic mutations or pharmacological intervention on the dynamics of asyn in the brains. We showed that *gba1* KO medaka displayed not only the dopaminergic (DA), noradrenergic (NA) and serotonergic neuronal cell loss, neuroinflammation, and ALP dysfunction, but also asyn accumulation in the brains [33]. *gba1* KO medaka are a useful model animal for investigating the pathological mechanisms underlying the CNS pathology in *GBA1*-related disorders.

In the present study, we generated *gba2* KO medaka and analyzed *gba2* single- and *gba1/gba2* double-KO (DKO) medaka to investigate the contributions of GBA2 to nGD. We found that the deletion of Gba2 did not rescue the CNS phenotypes of *gba1* KO medaka but resulted in the perturbation of sphingolipid metabolism and an increase in the amount of asyn. The present study provides novel insights into the pathological role of GBA2 in nGD and asyn accumulation in the brains.

## Methods

### Generation of *gba2* KO and *gba1/gba2* DKO medaka

The ethics statement and maintenance of medaka were described previously [33]. Medaka experiments were approved by the Animal Experiments Committee of Kyoto University and conducted in accordance with national guidelines. Medaka were maintained in an aquaculture system with recirculating water at 27 °C in a 14-h light/10-h dark cycle.

Medaka of the Kyoto-cab strain, a substrain of Cab, were used in this study. The generation and characterization of GBA1-deficient medaka were reported previously [33]. GBA2-deficient medaka were generated using a clustered regularly interspaced short palindromic repeats (CRISPR)/CRISPR-associated 9 (Cas9) system, as reported previously [34]. In brief, the cDNA sequence of medaka *gba2* was determined by reverse transcription-polymerase chain reaction and rapid amplification of cDNA ends. The medaka *gba2* gene consists of 18 exons encoding 858 amino acids. The crRNAs were designed using the CRISPR design tool (http://viewer.shigen.info/cgi-bin/crispr/crispr.cgi), and the following crRNA was used: 5′-GGAGGGCAAAGCACTGTCGGGGG-3′. The crRNA and tracrRNA were constructed by Fasmac Co. (Kanagawa, Japan). The Cas9 RNA was synthesized from pCS2 + hSpCas9 vector (Addgene #51815) using mMessage mMachine SP6 Kit (Thermo Fisher Scientific, Waltham, MA, USA). The RNA mixture was injected into single-cell-stage embryos. The injected founders (F_0_) were raised to sexual maturity and back-crossed with wild-type (WT) to generate F_1_s. The *gba*2 gene of F_1_s was sequenced, and novel heterozygous *gba2* mutation (*gba2*^+*/*−^) medaka with 21 bases deleted and 2 bases inserted into exon 5 were obtained (Additional file 1: Fig. S1). These deletions and insertions resulted in a frame shift mutation, leading to the deficiency in protein expression and enzymatic activity of Gba2 in the brain. Off-target candidates were searched for using the Medaka pattern match tool (http://viewer.shigen.info/medakavw/crisprtool/). No alterations were found in three off-target candidates located on exons.

*gba2*^+*/*−^ medaka were back-crossed with WT medaka at least five times and then crossed with GBA1-deficient medaka to create *gba1/gba2* DKO medaka. Medaka brains collected by surgery were directly snap-frozen in liquid nitrogen and then stored at − 80 °C until use. The *gba2*^+*/*−^ medaka and *gba2* KO medaka were used in the previously reported study [35].

### The GBA2 enzymatic activity assay

The measurement of GBA2 enzymatic activity was carried out as described previously [36, 37]. Medaka brains were homogenized in 200 µl deionized water and centrifuged at 500×*g* at 4 °C for 10 min. The supernatant was collected and centrifuged at 20,000×*g* at 4 °C for 20 min. The pellet was rinsed with 200 µl of 50 mM potassium phosphate buffer, pH 5.8 and centrifuged at 20,000×*g* at 4 °C for 15 min. This step was repeated twice. Next, 30 µl of 50 mM potassium phosphate buffer, pH 5.8 was added to the pellet, and the pellet was resuspended. The resulted suspension was used for the enzyme assay.

The reaction mixture contained 10 µl of the suspension and 20 µl of 4.5 mM 4-methlumbeliferyl β-d-glucopyranoside (Wako, #324-37411) in 100 mM citric acid and 200 mM disodium hydrogen phosphate buffer, pH 5.8. The reaction mixture was incubated at 37 °C for 60 min with or without 0.3 mM NB-DGJ (*N*-deoxygalactonojirimycin, #B690500; Toronto Research Chemicals Inc., Toronto, Canada). The reaction was terminated by adding 200 µl of 0.5 M sodium carbonate buffer at pH 10.7, and the fluorescence (excitation 55 nm and emission 460 nm) was measured by Fluoroskan Ascent FL (Thermo Fisher Scientific). The GBA2 enzymatic activity was measured as the GCase activity sensitive to NB-DGJ.

### Locomotor function analysis

The medaka locomotor function analysis was performed as described previously [33]. Medaka were transferred to a 20-cm-diameter tank filled with water to a depth of 2 cm at room temperature. After 5 min of rest, the free-swimming distance for 5 min was analyzed using an ethovision XT 5 software (Noldus, Leesburg, VA).

### Immunoblotting analysis

For the asyn, p62, LC3, neuron specific enolase (NSE) and β-actin analysis, the brains of medaka were homogenized in high-salt buffer containing 1% Triton X-100 (750 mM NaCl, 5 mM EDTA, 50 mM Tris–HCl, 1% [v/v] Triton X-100, pH 7.5). For the GBA2 analysis, the brains of medaka were homogenized in RIPA buffer (50 mM Tris–HCl, 0.15 M NaCl, 1% [v/v] Triton X-100, 0.1% [w/v] sodium dodecyl sulfate (SDS), 1% [v/v] sodium deoxycholate, pH 7.5). The homogenate was centrifuged at 20,400×*g* at 4 °C for 5 min. For SDS-soluble fractions, the pellet of asyn analysis was subsequently sonicated in SDS buffer (50 mM Tris–HCl, 2% SDS, pH 7.4) followed by centrifugation at 20,400×*g* at 4 °C for 5 min. The supernatant was collected, and the protein concentration was measured using a BCA protein assay kit (Pierce, Rockford, IL, USA). The supernatant was then mixed with sample buffer (1% [w/v] SDS, 12.5% [w/v] glycerol, 0.005% [w/v] bromophenol blue, 2.5% [v/v] 2-mercaptoethanol, 25 mM Tris–HCl, pH 6.6) and boiled at 95 °C for 10 min. The boiled samples containing 10 µg of protein or 0.75 µg for SDS-soluble fractions of protein were separated on 4–12% NuPAGE Bis–Tris Precast Gel (Thermo Fisher Scientific) or 10–20% SuperSep ^TM^ACE (FUJIFILM Wako Pure Chemical Corporation, Osaka, Japan) and transferred to polyvinylidene difluoride membranes using a Trans-Blot SD semi Dry Transfer Cell (Bio-Rad Laboratories Inc., Hercules, California, USA).

To detect medaka asyn, the membranes were treated with 4% (w/v) paraformaldehyde in phosphate-buffered saline (PBS) for 30 min at room temperature before being blocked with 5% skim milk [33, 38]. The following antibodies were used as the primary antibody: anti-β-actin (#A1978, 1:5000; Sigma-Aldrich Co., St. Louis, MO, USA), anti-LC3 (#PM036, 1:2000; MBL, Nagoya, Japan), anti-NSE (#M0873, 1:500; DAKO, Carpinteria, CA, USA), anti-medaka asyn (1:10,000) [33] and anti-p62 (#PM045, 1:500; MBL). Anti-medaka GBA2 antibody was raised against 377–395 amino acids of medaka GBA2 at Sigma-Aldrich Co. (1:1000). The membrane was incubated with anti-β-actin for 60 min at room temperature or with other primary antibodies for 1 day at 4 °C. Subsequent steps were performed according to the standard method using horseradish peroxidase-conjugated secondary antibodies (1:5000; Novus, Biologicals, Littleton, CO, USA) for 1 h at room temperature. The chemiluminescent signal was detected using an Amersham Imager 600 (GE Healthcare, Chicago, IL, USA).

### Confirmation of the cross-species reactivity of antibodies

To confirm the cross-species reactivity of the antibodies used in present study against medaka, the homology search for β-actin, LC3, NSE and p62 genes was performed (https://asia.ensembl.org/Oryzias_latipes/Info/Index), showing high homology of medaka β-actin, LC3, and NSE to the peptides used to raise each antibodies (93%, 93%, and 84%, respectively). Meanwhile, medaka p62 has only 45% homology to the peptide used to raise the p62 antibody. Therefore, we confirmed the cross-species reactivity of the antibody by overexpression of medaka p62 in cultured cells. The medaka *p62* cDNA was cloned into pcDNA3 and expressed in HEK293T cells. A strong signal was detected at the molecular weight close to that of medaka p62 in the lanes of medaka p62-overexpressing HEK293T cells and *gba1* KO medaka brains, but not in the lane of mock transfected cells (Additional file 2: Fig. S2). The following is the brief description about this experiment. The sequence of medaka *p62* cDNA was determined previously [33]. The full-length medaka *p62* cDNA was inserted into pcDNA3 to generate a medaka p62-expressing vector. Transfection of HEK293T cells (RIKEN Cell Bank, Tsukuba, Japan) with vector alone (pcDNA3-mock) or vector containing full-length medaka p62 (pcDNA3p62) was performed using polyethyleneimine MAX (#24765, Polysciences, Warrington, PA, USA) according to the manufacturer’s instructions. The cell lysates were obtained with RIPA buffer. The SDS-PAGE and chemiluminescent signal detection was performed as described above. The amount of loading protein was 5 μg for WT and *gba1* KO medaka brains and 0.05 μg and 0.25 μg for cell lysates, respectively.

### Immunohistochemical analysis

Paraffin sections were used for immunohistochemical analysis as reported previously [33]. Medaka brains were collected by surgery. The collected brains were fixed with 4% (w/v) paraformaldehyde in PBS at 4 °C for 1 day and stored in 70% ethanol until use. The fixed samples were dehydrated and embedded in paraffin using Surgipath FSC22 (Leica, Wetzlar, Germany), and sections were acquired using a Microm HM 325 (Thermo Fisher Scientific). The thickness of the sections was set at 20 µm for tyrosine hydroxylase (TH) -positive cell counting and 8 µm for other analysis. The following antibodies were used as the primary antibody: anti-TH (#MAB318, 1:1000; Merck Millipore, Burlington, MA) and anti-medaka asyn (1:2000) [33]. The sections were incubated with the primary antibody at 4 °C for 1 day after blocking with 4% skim milk. Histofine (#414322; Nichirei Bioscience Inc., Tokyo, Japan) was used as the secondary antibody for diaminobenzidine staining.

### Cell counting

The number of DA neurons in the middle diencephalon and NA neurons in the locus coeruleus were counted as previously described [33]. The numbers of TH-positive neurons with visible nuclei were counted under the microscope (Cx41; Olympus, Tokyo, Japan).

### Quantitative reverse transcription polymerase chain reaction (qRT-PCR)

RNA was isolated from medaka brains using Qiazol (QIAGEN). cDNA was synthesized using the PrimeScript RT reagent kit of Perfect Real Time (#RR037A; Takara, Kyoto, Japan). The quantification of cDNA was performed with the LightCycler 480 using LightCycler 480 SYBR GreenI Master (#04887352001; Roche Diagnostics, Mannheim, Germany). The following primer sets were used: TNF-α: 5′-ATTGGAGTGAAAGGCCAGAA-3′ and 5′-ACTAATTTGAGACCGCCACG-3′; β-actin: 5′-TCCACCTTCCAGCAGATGTG-3′ and 5′-AGCATTTGCGGTGGACGAT-3′; apolipoprotein E (ApoE)-b: 5′-GACGAGAGTTGGAGACCCTGA-3′ and 5′-ACTGGTGCTTGTGGTGATGG-3′; and asyn: 5′-ATGGACGCGTTAATGAAGGGTTT-3′ and 5′-TCAGTCATCGCTGTCTTCCT-3′.

### High performance liquid chromatography for the dopamine quantification

Medaka brains were homogenized in 100 µl of 0.1 M HClO_4_ containing 4 mM Na_2_S_2_O_5_ and 4 mM diethylenetriaminepentaacetic acid. The supernatant by centrifugation at 20,400×*g* for 5 min was used for measurement. High performance liquid chromatography (HPLC) was conducted with a mobile phase A (acetonitrile:methanol, 1000:25.9:62.9 [v/v/v], with 0.1 M phosphate, 0.05 M citrate, 4 mM sodium 1-heptanesulfonate and 0.1 mM EDTA, pH 3.0). Dopamine was detected with series coulometric detector (ESA, Inc., Chelmsford, MA, USA). Data were collected and analyzed on a CHROMELEONTM Chromatography Data Systems 6.40 (Dionex, Sunnyvale, CA, USA).

### The material for the sphingolipid profile analysis

β-d-Glucopyranosyl-(1 → 1)-*N*-lauroyl-d-*erythro*-sphingosine (GlcCer [d18:1-C12:0]), β-d-glucopyranosyl-(1 → 1)-d-*erythro*-sphingosine-*d*5 (GlcSph-*d*5), *N*-lauroyl-d-*erythro*-sphingosine (ceramide [d18:1-C12:0]) and d-*erythro*-sphingosine (C17 base) (d17:1-sphingosine) were purchased from Avanti Polar Lipids (Alabaster, AL, USA). For liquid chromatography (LC)-electrospray ionization tandem mass spectrometry (ESI–MS/MS), high-performance LC-grade acetonitrile and methanol were purchased from Thermo Fisher Scientific (Waltham, MA, USA), chloroform and distilled water were purchased from Kanto Chemical Co., Inc. (Tokyo, Japan), and ammonium formate was purchased from Sigma Aldrich, Japan.

### Lipid extraction for the sphingolipid profile analysis

The frozen tissue (approximately 5 mg) was homogenized, and total lipids were extracted with a chloroform:methanol (C:M) (2:1 [v/v], 5 ml) mixture spiked with 1 pmol/mg frozen tissue of GlcCer (d18:1-C12:0), GlcSph-*d*5, ceramide (d18:1-C12:0) and d17:1-sphingosine as internal standards. Extracts were dried under a flow of N_2_ gas and hydrolyzed for 2 h at room temperature in C:M (2:1 [v/v], 2 ml) containing 0.1 M KOH. The reaction mixture was neutralized with 7.5 µl of glacial acetic acid.

For the analysis of GlcCer, galactosylceramide (GalCer), GlcSph and galactosylsphingosine (GalSph: psychosine), the neutralized reaction mixture was subjected to Folch’s partition, and the lower phase was dried under a flow of N_2_ gas. For the analysis of ceramide and sphingosine, the neutralized reaction mixture was dried under a flow of N_2_ gas. The resulting lipid films were suspended in C:M (2:1, v/v) at a concentration of 100 µg frozen tissue/µl, and aliquots were subjected to LC–ESI–MS/MS.

### LC–ESI–MS/MS for the sphingolipid profile analysis

LC–ESI–MS/MS was performed on an LC system Nexera X2 (SHIMADZU, Kyoto, Japan) attached to a triple-quadrupole linear ion trap mass spectrometer (QTRAP4500; SCIEX, Tokyo, Japan). The LC–ESI–MS/MS datasets were analyzed with the MultiQuant™ (ver. 2.1) and Analyst (SCIEX) software programs. Target lipids were monitored in multiple reaction monitoring (MRM) mode using specific precursor-product ion pairs, as detailed in Additional file 3: Table S1. Peak areas were integrated and quantified relative to the associated internal standard.

GlcCer, GalCer, GlcSph and GalSph were analyzed as previously reported [15, 39, 40] by hydrophilic interaction chromatography (HILIC)-ESI–MS/MS with minor modifications. In brief, 100 µg frozen tissue/µl of the lipid extracts was diluted tenfold with mobile phase A (acetonitrile:methanol:formic acid, 97:2:1 [v/v/v], with 5 mM ammonium formate), and aliquots (10 µl) were applied to an Atlantis silica HILIC column (2.1 mm i.d. × 150 mm, particle size, 3 µm; Waters, Milford, MA) maintained at 40 °C. Samples were eluted at a flow rate of 0.15 ml/min using the following gradient of mobile phase B (methanol:water:formic acid, 89:9:1 [v/v/v], with 20 mM ammonium formate): 3.3 min, 0%; 13.4 min, 0–35% linear gradient; 1.3 min, 35–70% linear gradient; 3 min, 70% (washing step); 29 min, 0%, flow rate increased to 0.2 ml/min (equilibration); 1 min, 0%, flow rate decreased to 0.15 ml/min. The mass spectrometer was set to positive ion mode (ion spray voltage, 5500 V; curtain gas pressure, 30 psi; nebulizer gas pressure, 90 psi; heating gas pressure, 30 psi, temperature, 100 °C) using MRM detection for a targeted analysis. The quantitative values of GlcCer and GalCer with various chain lengths of fatty acids (C14:0, C16:0, C18:1, C18:0, C20:1, C20:0, C22:1, C22:0, C23:1, C23:0, C24:1, C24:0 and C26:1) were summarized.

Ceramide and d-*erythro*-sphingosine (C18 base) (sphingosine (C18 base)) were analyzed as previously reported [39] by reversed-phase liquid chromatography (RPLC)-ESI–MS/MS with minor modifications. The lipid extracts dissolved in C:M (2:1, v/v) were diluted tenfold with mobile phase B (M:W 85:15 [v/v], 5 mM ammonium acetate) and applied to an RP column (Luna C18(2) column; 2 mm i.d. × 250 mm, particle size, 3 μm; Phenomenex, Torrance, CA, USA) maintained at 36 °C and at a flow rate of 0.15 ml/min. The samples were then eluted with the following gradients of mobile phase A (methanol pure, 5 mM ammonium acetate): 2 min, 0%; 13 min, 0–100% linear gradient; 40 min, 100% (washing step); 15 min 0% (equilibration). The mass spectrometer was set to positive ion mode (ion spray voltage, 5500 V; curtain gas pressure, 20 psi; nebulizer gas pressure, 80 psi; heating gas pressure, 40 psi, temperature, 100 °C), utilizing either MRM detection for a targeted analysis. The quantitative values of ceramide with various chain lengths of fatty acids (C16:0, C18:0, C24:1 and C24:0) were summarized.

### Statistical analysis

For comparison of two groups, at two-tailed unpaired Student’s *t*-test was performed. An F test was performed to evaluate the differences in variances. For comparison of three or more groups, one-way ANOVA with Tukey’s post-hoc test or Newman-Keuls multiple comparison test was performed. A Brown–Forsythe test was performed to evaluate the differences in variances. Differences with p values of less than 0.05 were considered significant. Statistical calculations were performed with GraphPad Prism Software (GraphPad) Version5.0 and 7.04.

## Results

### *gba2* KO medaka show no apparent phenotypes

First, we attempted to create *gba2* KO medaka using a CRISPR/Cas9 system. We successfully generated *gba2* nonsense mutant medaka via the deletion of 21 bases and insertion of 2 bases in exon 5, which resulted in a frame shift mutation (Additional file 1: Fig. S1).

There were 15 off-target candidates of crRNA used in this study, among which 3 were located on exons (Additional file 4: Table S2). We conducted direct sequencing and confirmed no mutations around these three off-target candidates in medaka carrying heterozygous mutations of the *gba2* gene.

**Fig. 1:**
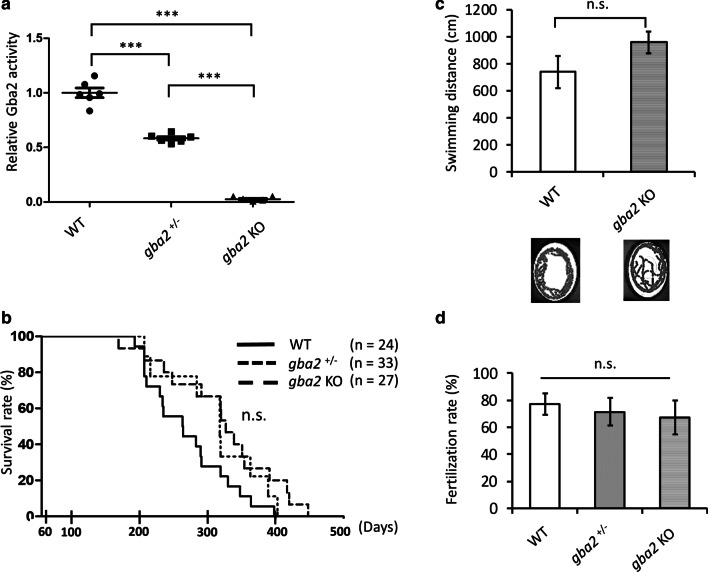
Phenotypes of *gba2* KO medaka. **a** The Gba2 enzymatic activity in the brains of medaka. The Gba2 activity was almost eliminated in *gba2* KO medaka and reduced by 42% in *gba2*^+*/*−^ medaka (n = 6 for each genotype). A one-way ANOVA with Tukey's multiple comparison test was performed. **b** A Kaplan–Meier plot showed no significant differences in the survival among genotypes (n = 24–33 for each genotype). A Log-rank (Mantel-Cox) test was performed. **c** Analysis of the spontaneous swimming movement. *gba2* KO medaka showed a normal locomotor function compared with WT medaka at 6 mpf (n = 6 for each genotype). A two-tailed unpaired Student’s *t*-test was performed. The bottom images are representative movement tracks for each genotype. **d** Fertilization rate analysis showed no marked differences among genotypes at 6 pmf (n = 3 for each group). A one-way ANOVA with Tukey's multiple comparison test was performed. *n.s.* not significant, ***p < 0.001. The bars indicate the mean ± SEM

We then analyzed the phenotypes of *gba2* KO medaka after heterozygous *gba2* mutant medaka were backcrossed with WT medaka more than five times. *gba2* KO medaka showed almost no enzymatic activity or protein expression of Gba2 in the brains, and *gba2*^+*/*−^ medaka showed ~ 58% of enzymatic activity and ~ 42% of protein expression level compared with WT medaka (Fig. 1a and Additional file 5: Fig. S3). The lifespan of both *gba2* KO and *gba2*^+*/*−^ medaka were the same as that of WT medaka (Fig. 1b).

We next examined their locomotor function, as the loss of GBA2 causes locomotor dysfunction in mice and zebrafish [25, 29]. In contrast to GBA2-deficient mice and zebrafish, *gba2* KO medaka showed a normal locomotor function at 6 mpf (Fig. 1c). Such differential effects of GBA2 loss between medaka and mice are seen also in spermatogenesis, as *Gba2* KO mice show infertility due to morphological abnormality of sperm, called globozoospermia, while *gba2* KO medaka showed a normal fertilization rate (Fig. 1d). In summary, *gba2* KO medaka did not show any apparently abnormal phenotypes.

### Deletion of Gba2 in *gba1* KO medaka does not rescue the CNS phenotype

**Fig. 2:**
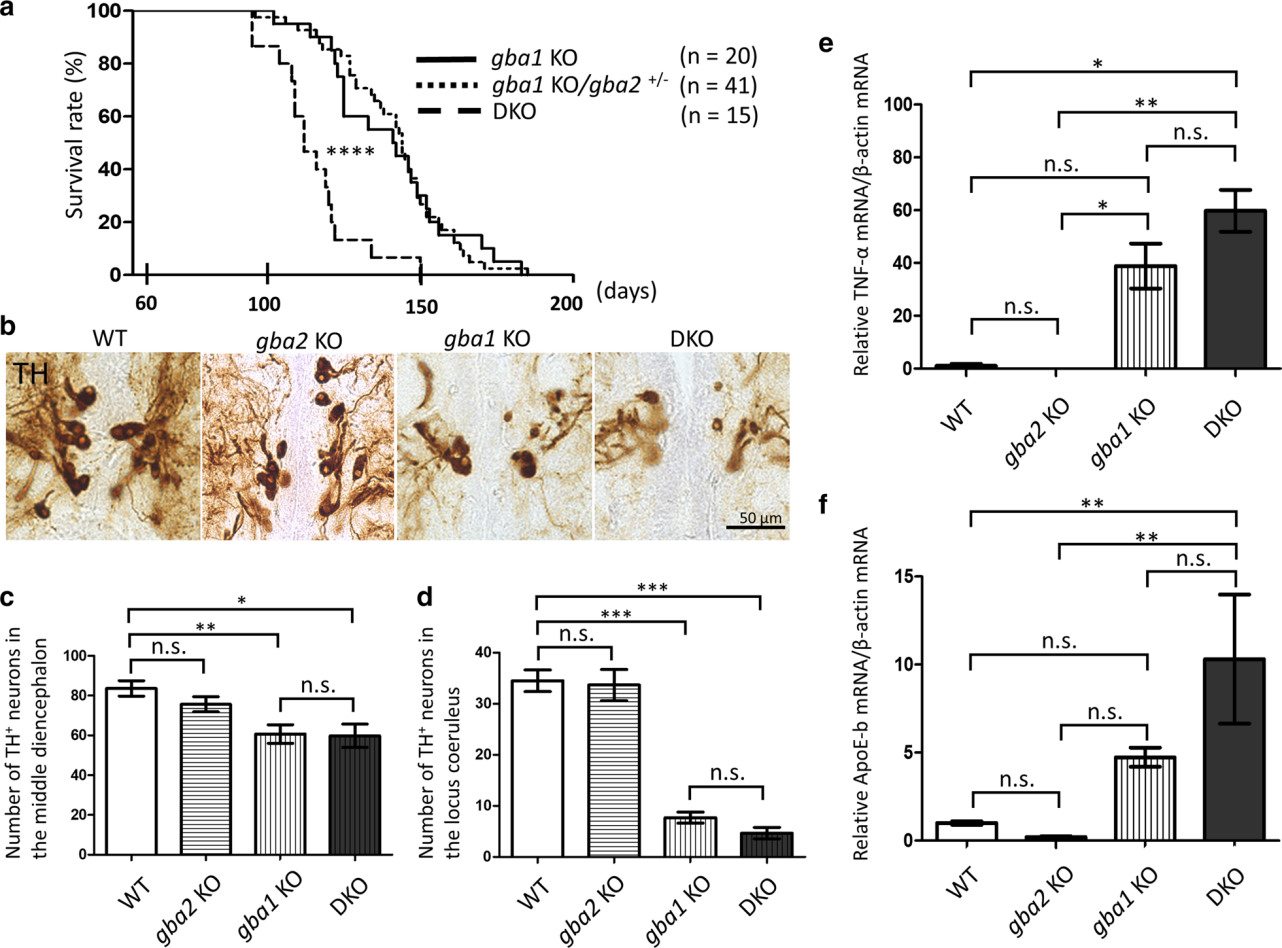
Deletion of Gba2 in *gba1* KO medaka did not rescue their phenotypes. **a** A Kaplan–Meier plot showed that the deletion of Gba2 in *gba1* KO medaka shortened the lifespan (n = 15–41 for each genotype). A Log-rank (Mantel-Cox) test was performed. **b** Representative TH immunohistochemical staining of the middle diencephalic dopaminergic neurons in WT, *gba1* KO, and *gba1/gba2* DKO medaka at 3 mpf. **c** The number of TH-positive (TH^+^) neurons in the middle diencephalon at 3 mpf. The number of TH^+^ neurons was decreased in *gba1/gba2* DKO medaka compared with WT medaka but did not markedly differ between *gba1/gba2* DKO and *gba1* KO medaka (n = 6–10 for each genotype). A one-way ANOVA with Tukey's multiple comparison test was performed. **d** The number of TH^+^ neurons in the locus coeruleus at 3 mpf. The number of TH^+^ neurons was decreased in *gba1/gba2* DKO medaka compared with WT medaka but did not differ markedly between *gba1/gba2* DKO and *gba1* KO medaka (n = 4–10 for each genotype). A one-way ANOVA with Tukey's multiple comparison test was performed. **e** qRT-PCR analysis of TNF-α mRNA in the brains of 3 mpf medaka. TNF-α mRNA levels were normalized to β-actin mRNA. The TNF-α mRNA levels did not differ markedly between *gba1/gba2* DKO and *gba1* KO medaka (n = 6 for each genotype). Dunn’s multiple comparisons test was performed. **f** qRT-PCR analysis of ApoE-b mRNA in the brains of 3 mpf medaka. The ApoE-b mRNA levels were normalized to β-actin mRNA. The amount of ApoE-b mRNA was increased in *gba1/gba2* DKO medaka compared with WT medaka but did not differ markedly between *gba1/gba2* DKO and *gba1* KO medaka (n = 6 for each genotype). A one-way ANOVA with Tukey's multiple comparison test was performed. *n.s.* not significant, ****p < 0.0001. The bars indicate mean ± SEM

The compensatory changes of GBA2 in GBA1 deficiency remain unclear given the previous studies showing conflicting data [18, 28, 41–43]. Therefore, we examined Gba2 protein expression levels and enzymatic activity in the brains of WT and GBA1 deficient medaka, showing no differences in protein expression levels or enzymatic activity among the *gba1* genotypes (Additional file 6: Fig. S4a and b). To examine whether or not GBA2 functioned as a novel factor affecting nGD, we crossed *gba2* mutant medaka and *gba1* mutant medaka to generate *gba1/gba2* DKO medaka. Recent studies have reported that the deletion of GBA2 in a GD1 mouse model and a NPC mouse model improved the phenotypes [30, 31]. However, *gba1/gba2* DKO medaka showed a shorter lifespan than *gba1* KO medaka (Fig. 2a). In addition, *gba1/gba*2 DKO medaka did not show improvement of abnormal swimming movement seen in *gba1* KO medaka [33] (Additional files 7, 8, 9, 10: video S1–4).

We previously reported that *gba1* KO medaka showed the DA, NA and serotonergic neuronal cell loss, increased inflammatory cytokine levels and microgliosis similar to nGD patients [33]. We then evaluated the neuronal cell loss by counting the number of TH-positive neurons in the middle diencephalon (DA neurons) and locus coeruleus (NA neurons). The numbers of both DA and NA neurons were decreased in *gba1/gba2* DKO medaka, similar to *gba1* KO medaka at 3 months mpf (Fig. 2b–d). The amount of DA in the brains showed trend towards decrease in *gba1* KO and *gba1/gba2* DKO medaka albeit not reaching statistical significance (Additional file 11: Fig. S5). We also examined the neuroinflammation in *gba1/gba2* DKO medaka. The mRNA expression of tumor necrosis factor (TNF)-α was not markedly different between *gba1/gba2* DKO and *gba1* KO medaka at 3 mpf (Fig. 2e). We evaluated the expression of mRNA encoding ApoE-b, a marker for microglia in teleost fish [33, 44], and confirmed a similar expression between *gba1/gba2* DKO and *gba1* KO medaka at 3 mpf (Fig. 2f). *gba1*/*gba2* DKO medaka also showed the Gaucher cell-like cells which had been seen in *gba1* KO medaka [33] (Additional file 12: Fig. S6). These results suggested that the deletion of Gba2 does not rescue any CNS phenotypes of *gba1* KO medaka.

### Perturbation of sphingolipid metabolism in the brains of *gba1* and *gba2* mutant medaka

**Fig. 3:**
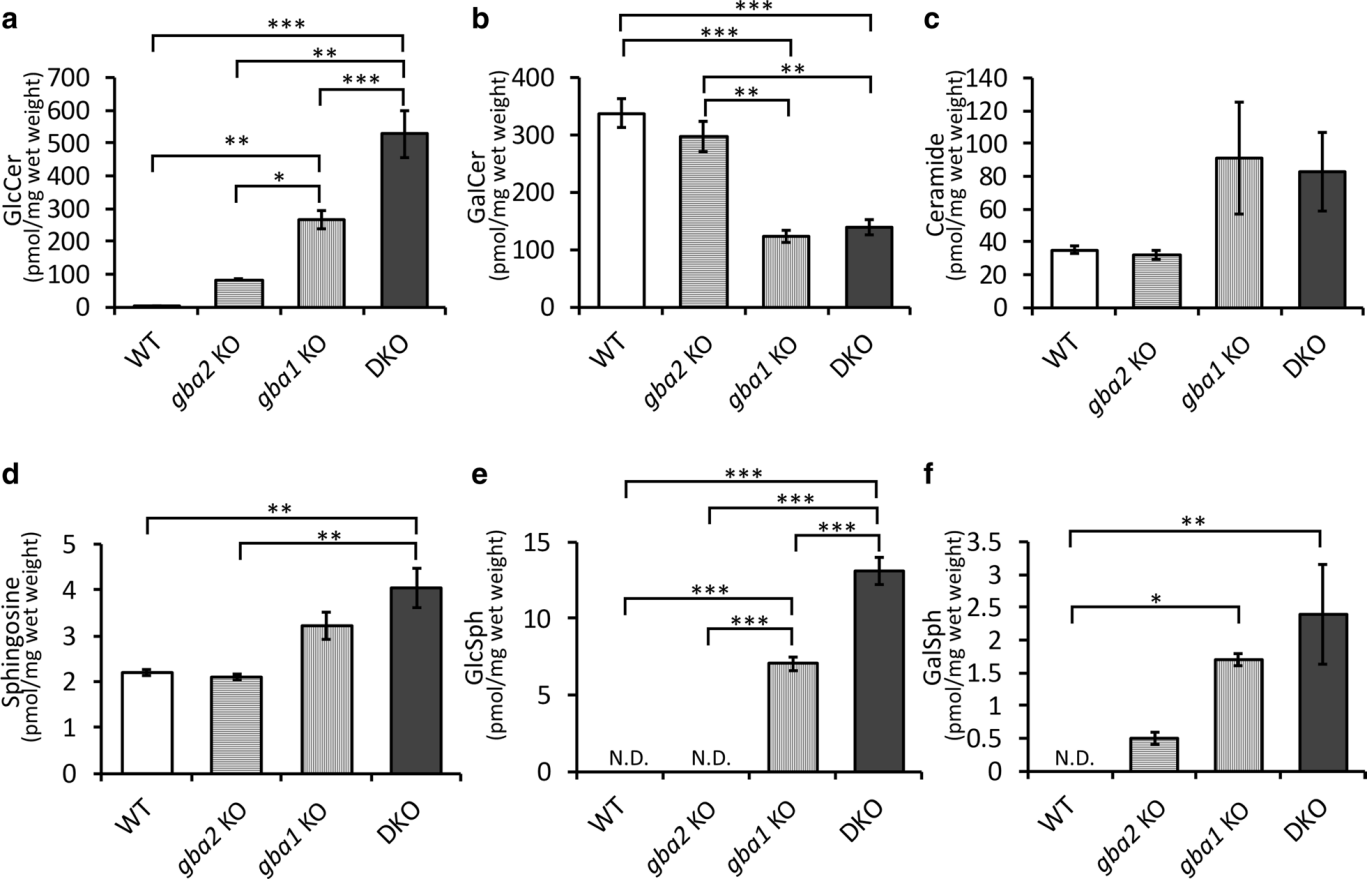
The amount of sphingolipids was altered in the brains of *gba1* and *gba2* mutant medaka. **a** The amount of GlcCer was increased in *gba1/gba2* DKO medaka compared with *gba1* KO medaka (n = 3 for each genotype). **b** The amount of GalCer did not differ markedly between *gba1* KO and *gba1/gba2* DKO medaka (n = 3 for each genotype). **c** The amount of ceramide did not differ among genotypes (n = 3 for each genotype). **d** The amount of sphingosine did not differ markedly between *gba1* KO and *gba2* KO medaka (n = 3 for each genotype). **e** The amount of GlcSph was increased in *gba1/gba2* DKO compared with *gba1* KO medaka (n = 3 for each genotype). **f** The amount of GalSph was increased in *gba1* KO and *gba1/gba2* DKO medaka compared with WT medaka (n = 3 for each genotype). A one-way ANOVA with Tukey's multiple comparison test was performed. N.D.: not detected (below the detection threshold). *n.s.* not significant, *p < 0.05, **p < 0.01 and ***p < 0.001. The bars indicate the mean ± SEM

To examine why Gba2 deletion did not rescue the CNS phenotypes of *gba1* KO medaka, we analyzed the sphingolipid profiles in the medaka brains at 3 mpf using LC–ESI–MS/MS which can measure GlcCer and GalCer separately. We previously reported that *gba1* KO medaka showed the accumulation of GlcCer, similar to GBA1-deficient human and mice [33]. The sphingolipid analysis showed that the amounts of GlcCer and glucosylsphingosine (GlcSph) were further increased in *gba1/gba2* DKO medaka compared with *gba1* KO medaka (Fig. 3a and e). The amount of GlcCer tended to be increased in *gba2* KO medaka compared with WT medaka (Fig. 3a and Additional file 13: Table S3). While the amount of ceramide did not differ among the genotypes (Fig. 3c), that of GalCer was decreased in the absence of Gba1, irrespective of the *gba2* genotype (Fig. 3b). In contrast, GalSph was increased in *gba1* KO and *gba1/gba2* DKO medaka (Fig. 3f). While the amount of GalSph was below the detection threshold in WT medaka, it was detected in *gba2* KO and *gba1*^±^ medaka (Fig. 3f and Additional file 13: Table S3). GalSph is a substrate of galactocerebrosidase (GALC) and is believed to be the pathological sphingolipid involved in Krabbe’s disease (KD) [45]. The amount of sphingosine, which was reported to have cellular toxicity in GD1 [30], was not decreased in *gba1/gba2* DKO medaka compared with *gba1* KO medaka (Fig. 3d).

In summary, compared with *gba1* KO medaka, *gba1/gba2* DKO medaka showed a significant increase in GlcCer and GlcSph, and an increasing trend in sphingosine and GalSph in the brains. Although *gba2* KO medaka did not show significant increase in the amount of GlcCer and GalSph when comparing all groups, it reached statistical significance when compared with WT medaka (Additional file 13: Table S3).

### The impact of Gba2 on asyn accumulation

**Fig. 4:**
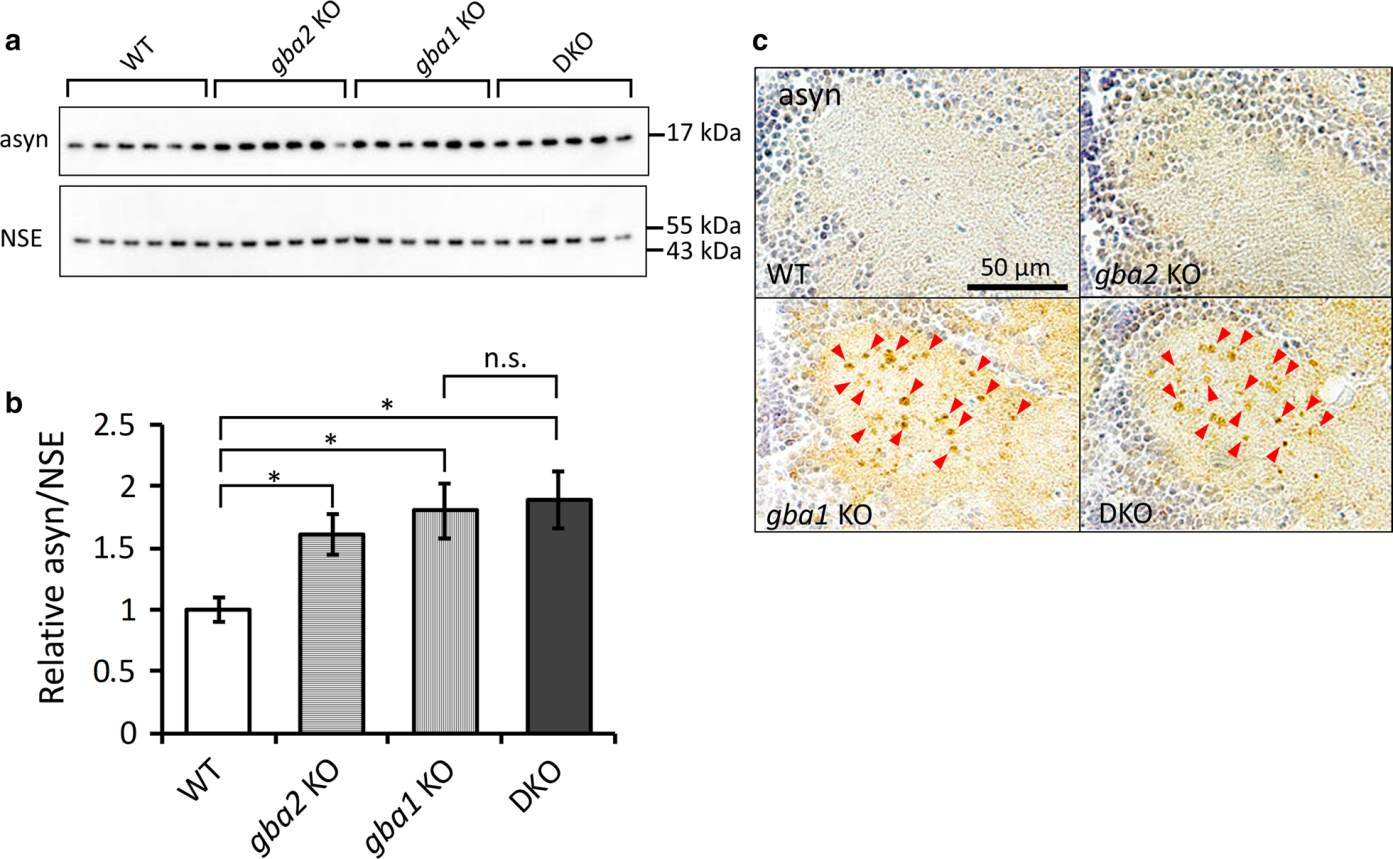
Gba1 and Gba2 deficiency caused asyn accumulation in the medaka brains. **a**, **b** Immunoblot analysis of asyn in the brains at 3 mpf. Asyn was increased in the *gba1* KO, *gba2* KO and *gba1/gba2* DKO medaka compared with WT medaka (n = 6 for each genotype). NSE was used as a loading control. A one-way ANOVA Newman-Keuls multiple comparison test was performed. The bars indicate the mean ± SEM. *n.s.* not significant, *p < 0.05, **c** Asyn immunohistochemical staining of the diencephalon at 3 mpf. Asyn accumulations (arrowheads) were observed in *gba1* KO and *gba1/gba2* DKO medaka but not in *gba2* KO medaka or WT medaka

Recent studies have suggested that the perturbation of sphingolipid metabolism causes conformational changes in asyn, resulting in asyn accumulation [16, 18, 46]. *gba1* KO medaka showed the perturbation of sphingolipid metabolism accompanied by asyn accumulation in axonal swellings containing autophagosomes, which we previously determined by electron microscopic analysis [33]. The increase in asyn was seen in the Triton X-soluble fractions, but not in SDS-soluble fractions (Additional file 14: Fig. S7). Compared with WT medaka, *gba2* KO medaka showed an increased amount of asyn without any asyn-positive abnormal structures like axonal swellings seen in *gba1* KO medaka (Fig. 4a–c). *gba1/gba2* DKO medaka showed the similar phenotypes to those of *gba1* KO medaka (Fig. 4a–c). The qRT-PCR for *asyn* mRNA showed no increase in *asyn* mRNA expression levels in *gba2* KO medaka (Additional file 15: Fig. S8). This result suggests that asyn accumulation seen in *gba2* KO medaka was the pathological process after the translation.

### ALP dysfunction observed in *gba1* KO medaka but not in *gba2* KO medaka.

**Fig. 5:**
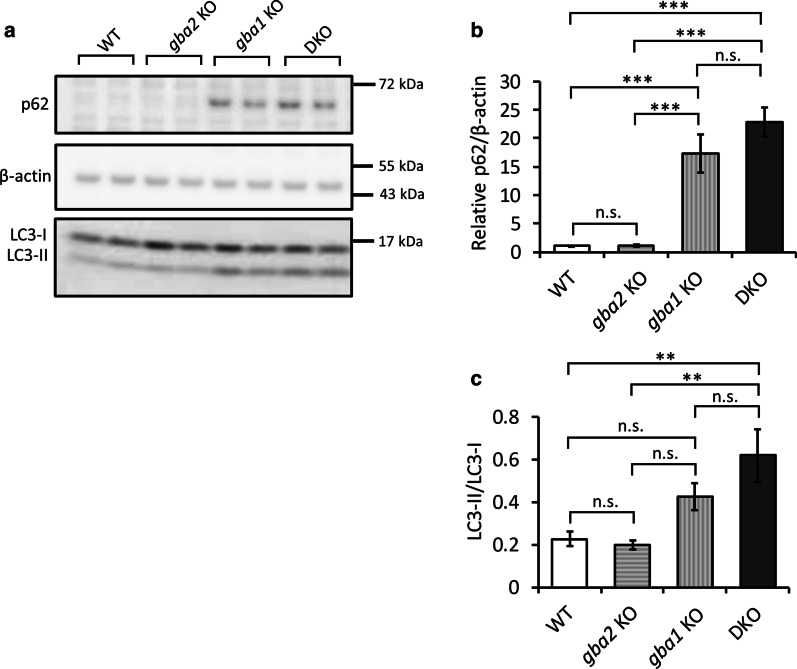
Autophagic dysfunction in *gba1* KO and *gba1/gba2* DKO medaka, but not in *gba2* KO medaka. **a**, **b** Immunoblot analysis of p62 in the brains at 3 mpf. The expression of p62 was increased in the *gba1/gba2* DKO and *gba1* KO medaka but not in *gba2* KO medaka (n = 6 for each genotype). **a**, **c** Immunoblot analysis of LC3-I and LC3-II in the brains at 3 mpf. The LC3-II/I ratio was increased in the *gba1/gba2* DKO and *gba1* KO medaka compared with the WT and *gba2* KO medaka (n = 6 for each genotype). A one-way ANOVA with Tukey's multiple comparison test was performed. *n.s.* not significant, **p < 0.01, and ***p < 0.001. The bars indicate the mean ± SEM

Perturbation of the sphingolipid metabolism may be one of the causes of the asyn accumulation in the brains of *gba2* KO medaka. Because both ALP dysfunction and the perturbation of the sphingolipid metabolism presumably leads to asyn accumulation in *gba1* KO medaka [33], we also examined the autophagic function in *gba2* KO medaka. p62 acts as a linker between microtubule-associated protein 1 light chain 3 (LC3) and poly-ubiquitinated proteins. The expression of p62 correlates with the autophagic function, while the LC3-II/I ratio correlates with the accumulation of autophagosomes [47]. The expression of p62 and the LC3-II/I ratio were not markedly different in the brains of *gba2* KO medaka from WT (Fig. 5a–c). We assumed that ALP was intact in *gba2* KO medaka in contrast to *gba1* KO medaka.

## Discussion

This study revealed that the deletion of Gba2 in *gba1* KO medaka did not rescue the phenotypes, including the altered sphingolipid metabolism and asyn accumulation in the brains. However, GBA2 represents a novel factor affecting asyn accumulation because *gba2* KO medaka showed an increased amount of asyn in the brains, which was presumably caused by the perturbation of sphingolipid metabolism.

In GD, the accumulation of substrates of GBA1, GlcCer and GlcSph, is thought to play a central role in disease pathogenesis. At present, substrate reduction therapy to reduce these sphingolipids is available for GD1. Although the mechanisms by which *GBA1* mutations cause PD are not fully understood yet, the following evidence suggests that a reduced GBA1 enzymatic activity is responsible for the increased risk of developing PD: (1) There is a negative correlation between GBA1 activity and asyn accumulation [17, 48]; (2) heterozygous *GBA1* mutations with lower enzymatic activities confer a higher risk of developing PD than those with higher enzymatic activities [49]; (3) GBA1 overexpression ameliorates asyn accumulation in a PD mouse model [50]. Furthermore, previous studies have shown that a brain-penetrating GlcCer synthase inhibitor and a GBA1 chaperone alleviated pathological asyn accumulation in the brains of PD mouse models [51, 52]. These findings suggest that decreased GBA1 enzymatic activity and the resultant substrate accumulation lead to increased risk of developing PD.

In the present study, we focused on another glucocerebrosidase, GBA2, as a novel factor affecting nGD, based on the findings of a previous study that the deletion of GBA2 ameliorated the visceral phenotypes of a GD1 mouse model [30]. However, contrary to our expectation, deletion of Gba2 did not rescue any CNS phenotypes of *gba1* KO medaka. In the previous study using the GD1 mouse model, the deletion of GBA2 was presumed to reduce the amount of sphingosine, thereby ameliorating the phenotypes of GD1 mice [30]. However, in the present study, the analysis of the sphingolipids profile revealed that the deletion of Gba2 in *gba1* KO medaka further tended to increase sphingosine in the brains, albeit without statistical significance. One possible explanation is that the *gba1* KO medaka is a complete deletion of Gba1, whereas GBA1 is only deleted in hematopoietic and mesenchymal lineage cells in *Mx1-Cre* (+): GD1 mice [30]. Therefore the effect of the GBA2 deletion on sphingolipid metabolism may be different between *Mx1-Cre* (+): GD1 mice and *gba1* KO medaka. Another possible explanation is the existence of an alternative pathway that produces sphingosine other than GBA1 and GBA2; human, rats and medaka, but not mice, possess GBA3. GBA3 hydrolyzes GlcCer and GlcSph to produce sphingosine in *gba1/gba2* DKO medaka but not in *gba1/gba2* DKO mice. This pathological effect of GBA3 is supported by a report that GBA3 does not affect the pathology of GD1, whereas GD1 patients with GBA3 deficiency showed a tendency to have a milder disease severity [53]. Thus, the failure to rescue the phenotypes of *gba1* KO medaka by Gba2 deletion may be relevant to human patients having *GBA1/GBA2* mutations. Moreover, it also needs to be considered that most patients with nGD have some residual GBA1 activity unlike *gba1* KO medaka. The effect of GBA2 deletion in other animal models of nGD with residual GBA1 activity should be examined in future studies.

**Fig. 6:**
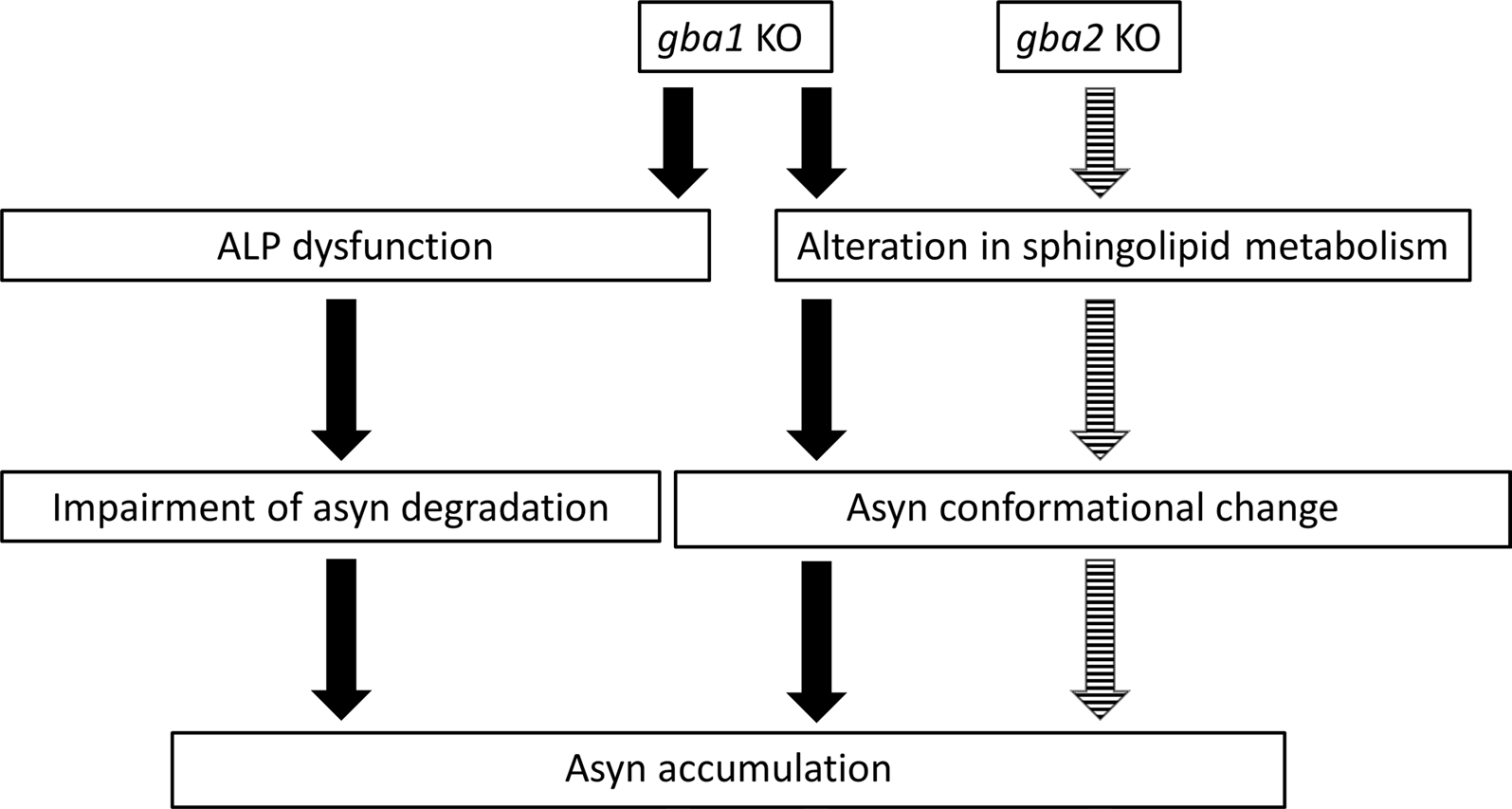
Proposed mechanisms of deletion of Gba1 and Gba2 leading to asyn accumulation. Deletion of Gba1 and Gba2 induces changes in the sphingolipid metabolism. These changes might induce conformational changes in asyn, resulting in asyn accumulation. Furthermore, the deletion of Gba1 causes autophagic dysfunction, resulting in the impairment of asyn degradation and leading to asyn accumulation. The solid and striped arrows indicate the downstream pathways of *gba1* KO and *gba2* KO, respectively

The present study reports, for the first time, the extensive sphingolipids profile in the brains of *GBA2* KO animals. Compared with WT medaka, the amounts of GlcCer and GalSph were found to be significantly increased in *gba2* KO medaka. Accumulating evidence has shown that metabolic abnormalities of sphingolipids are closely associated with the formation of asyn pathology [16, 17, 46, 54]. Under physiological conditions, asyn maintains an equilibrium state between free monomeric and membrane-bound multimeric conformation, which can be disrupted by sphingolipid metabolic perturbation [54–56]. Previous studies demonstrated that GlcCer converts the physiological asyn conformers to assembly-state pathological asyn, leading to its accumulation in cultured cells [17, 46]. In addition, a recent report indicated that GalSph also promotes asyn aggregation [57, 58]. Based on these previous studies, an increased amount of GlcCer and/or GalSph presumably causes asyn accumulation in the brains of *gba2* KO medaka. One of the biggest differences in asyn accumulation between *gba1* KO medaka and *gba2* KO medaka was observed by histological analyses. asyn accumulation was localized in axonal swellings in *gba1* KO medaka [33], whereas subcellular localization of asyn accumulation in *gba2* KO medaka could not be determined by immunohistochemistry (Fig. 4c). This is probably because asyn condensed in the axonal swellings in *gba1* KO medaka with ALP dysfunction, whereas asyn diffusely accumulated in *gba2* KO medaka. To our knowledge, the present study is also the first report showing the quantitative changes in GalSph in the brains of heterozygous *GBA1* mutant animals. Although its synthetic pathway is unclear, GalSph might play some pathological role in *GBA1*-PD. Based on the findings of the present and previous studies, we have proposed pathological mechanisms of asyn accumulation under *gba1* or *gba2* KO backgrounds (Fig. 6). Interestingly, it was reported that the GBA2 activity declines with aging in the mouse brain [59]. Given the asyn accumulation in *gba2* KO medaka, as shown in the present study, GBA2 may be a novel factor affecting asyn accumulation in the brains. Further studies are required to determine whether the perturbation of sphingolipid metabolism directly causes asyn accumulation in *GBA2* KO animals and how the age-dependent decline in GBA2 activity contributes to asyn accumulation. It should be noted there is no report that Miglustat (N-butyldeoxynojirimycin), an orphan drug for type 1 GD disease or NPC, which inhibits GBA2 activity as well as glucosylceramide synthase, induced parkinsonism [37, 43].

One limitation of the present study is that we failed to detect asyn in the Triton X-insoluble, SDS-soluble fractions in any genotypes partly because of small brains of medaka. Another limitation is that we did not detect pathological asyn aggregates on brain sections due to the lack of antibodies that recognize pathological medaka asyn, such as misfolded or phosphorylated forms of asyn. The other limitation is that we saw a limited synergistic effect on asyn accumulation in *gba1/gba2* DKO medaka compared with *gba*1 KO and *gba2* KO medaka (Fig. 4b), which seems against the proposed mechanisms (Fig. 6). One possibility is a ceiling effect of Gba1 and Gba2 deficiency on asyn accumulation, but we don’t have any evidence to support it.

In conclusion, the present study demonstrated that Gba2 deletion does not rescue the CNS phenotypes of *gba1* KO medaka. *gba2* KO presumably increases the amount of asyn in the medaka brain, through sphingolipid metabolic perturbation. Further studies are required to determine whether GBA2 could be a therapeutic target of α-synucleinopathy.

## Supplementary Information

**Additional file 1: Figure S1.** Genome structure of medaka GBA2 and mutations induced by CRISPR/Cas9. The red boxes represent exons, and the Greek numbers indicate the exon number. Medaka GBA2 consists of 2577 bases and 858 amino acids. The deletion of 21 bases and insertion of 2 bases in exon 5 of *gba2* led to a nonsense mutation. The underlined part represents the used crRNA sequence to generate the *gba2* KO medaka in this study.

**Additional file 2: Figure S2.** Confirmation of the cross-species reactivity of the p62 antibody. A strong signal was detected at the molecular weight close to that of medaka p62 in the lanes of medaka p62-overexpressing HEK293T cells and *gba1* KO medaka brains, but not in the lane of mock transfected cells.

**Additional file 3: Table S1.** Analytical conditions used for the analysis by MRM methods.

**Additional file 4: Table S2.** Off-target candidates of the crRNA used to generate *gba2* KO medaka in the present study. These candidates were identified with the Medaka pattern match tool (http://viewer.shigen.info/medakavw/crisprtool/). Three out of the 15 candidates were found in exons.

**Additional file 5: Figure S3.** Gba2 protein expression in *gba2* mutant medaka. Immunoblot analysis showed almost no expression of Gba2 protein in the brains of *gba2* KO medaka at 3 mpf. *gba2*^+*/*−^ medaka showed ~ 42% of Gba2 expression level compared with WT. (n = 4 for each genotype). n.s.: not significant, **p < 0.01 and ***p < 0.001 (a one-way ANOVA with Tukey's multiple comparison test). The bars indicate the mean ± SEM.

**Additional file 6: Figure S4.** Gba2 protein expression and enzymatic activity in *gba1* mutant medaka. (a) Immunoblot analysis of Gba2 in the brains of *gba1* mutant medaka at 3 mpf. The Gba2 protein expression did not significantly differ among genotypes (n = 4 for each genotype). (b) The Gba2 enzymatic activity of *gba1* mutant medaka at 3 mpf. The Gba2 activity did not significantly differ among genotypes (n = 8 for each group). A one-way ANOVA with Tukey's multiple comparison test was performed. n.s.: not significant. The bars indicate the mean ± SEM.

**Additional file 7: Video S1.** Swimming movement of WT medaka at 3 mpf.

**Additional file 8: Video S2.** Swimming movement of *gba2* KO medaka at 3 mpf.

**Additional file 9: Video S3.** Swimming movement of *gba1* KO medaka at 3 mpf.

**Additional file 10: Video S4.** Swimming movement of *gba1/gba2* DKO medaka at 3 mpf.

**Additional file 11: Figure S5.** Dopamine measurement of the brains by high performance liquid chromatography. Dopamine measurement of the brains was conducted at 3mpf. The data showed trend towards decrease in the amount of dopamine in the brains of *gba1* KO and *gba1/gba2* DKO medaka albeit not reaching statistical significance (n = 4 for each genotype). A one-way ANOVA with Tukey's multiple comparison test was performed. n.s.: not significant.

**Additional file 12: Figure S6.** Gaucher cell-like cells appeared in *gba1/gba2* DKO as well as *gba1* KO medaka brains. Hematoxylin and eosin staining showed abnormal cells in the periventricular gray zone of the optic tectum in *gba1/gba2* DKO and *gba1* KO medaka but not in the WT or *gba2* KO medaka (arrows). Enlarged images of Gaucher cell-like cells containing large vacuoles are shown in the insets (arrowheads). The representative Gaucher cell-like cells are circled in dot red line.

**Additional file 13: Table S3.** The sphingolipid profiles in each genotype. The amount of each sphingolipid in each genotype was compared with that in WT (n = 3 for each genotype). N.D.: not detected (below the detection threshold). n.s.: not significant, *p < 0.05, **p < 0.01, ***p < 0.001 and ****p < 0.0001 (a two-tailed unpaired Student’s *t*-test).

**Additional file 14: Figure S7.** Expression of asyn in Triton X-insoluble, SDS-soluble fractions. Expression of asyn was not detected in Triton X-insoluble, SDS-soluble fractions (shown “insoluble” in the figure). The immunoblot data of the Triton X-soluble (shown “soluble” in the figure) is the same as Fig. 4a.

**Additional file 15: Figure S8.** *gba2* mRNA expression in brains measured by qRT-PCR. Measurement of asyn mRNA expression in the brains was conducted at 3 mpf. (n = 4 for each genotypes). A one-way ANOVA with Tukey's multiple comparison test was performed. n.s.: not significant.

## Data Availability

The datasets used and analyzed during the current study are available from the corresponding author on reasonable request.

## Abbreviations

ApoE: Apolipoprotein E
asyn: Alpha synuclein
Cas9: CRISPR-associated 9
Ceramide [d18:1-C12:0]: *N*-Lauroyl-d-*erythro*-sphingosine
C:M: Chloroform:methanol
CNS: Central nervous system
CRIPSR: Clustered regularly interspaced short palindromic repeats
DA: Dopaminergic
DKO: Double knockout
ESI–MS/MS: Electrospray ionization tandem mass spectrometry
GalCer: Galactosylceramide
GalSph: Galactosylsphingosine
GALC: Galactosylceramidase
GCase: Glucocerebrosidase
GlcCer: Glucosylceramide
GlcCer (d18:1-C12:0): β-d-glucopyranosyl-(1 → 1)-*N*-lauroyl-d-*erythro*-sphingosine
GlcSph: Glucosylsphingosine
GlcSph-*d*5: β-d-glucopyranosyl-(1 → 1)-d-*erythro*-sphingosine-*d*5
GD: Gaucher’s disease
HILIC: Hydrophilic interaction chromatography
HPLC: High performance liquid chromatography
KD: Krabbe’s disease
KO: Knockout
LC: Liquid chromatography
LC3: Microtubule-associated protein 1 light chain 3
mpf: Months post fertilization
MRM: Multiple reaction monitoring
NA: Noradrenergic
NB-DGJ: *N*-Deoxygalactonojirimycin
NSE: Neuron specific enolase
nGD: Neuronopathic Gaucher’s disease
NPC: Niemann-Pick Type C
PBS: Phosphate-buffered saline
PD: Parkinson’s disease
qRT-PCR: Quantitative reverse transcription polymerase chain reaction
RPLC: Reversed-phase liquid chromatography
SDS: Sodium dodecyl sulfate
d17:1-Sphingosine: d-*erythro*-sphingosine (C17 base)
Sphingosine (C18 base): d-*erythro*-sphingosine (C18 base)
TH: Tyrosine hydroxylase
TNF: Tumor necrosis factor
WT: Wild type

## References

[1] Stirnemann J, Belmatoug N, Camou F, Serratrice C, Froissart R, Caillaud C (2017). A review of Gaucher disease pathophysiology, clinical presentation and treatments. Int J Mol Sci.

[2] Grabowski GA (2008). Phenotype, diagnosis, and treatment of Gaucher's disease. Lancet (London, England).

[3] Mitsui J, Mizuta I, Toyoda A, Ashida R, Takahashi Y, Goto J (2009). Mutations for Gaucher disease confer high susceptibility to Parkinson disease. Arch Neurol.

[4] Nalls MA, Duran R, Lopez G, Kurzawa-Akanbi M, McKeith IG, Chinnery PF (2013). A multicenter study of glucocerebrosidase mutations in dementia with Lewy bodies. JAMA Neurol.

[5] Sidransky E, Nalls MA, Aasly JO, Aharon-Peretz J, Annesi G, Barbosa ER (2009). Multicenter analysis of glucocerebrosidase mutations in Parkinson's disease. N Engl J Med.

[6] Alcalay RN, Dinur T, Quinn T, Sakanaka K, Levy O, Waters C (2014). Comparison of Parkinson risk in Ashkenazi Jewish patients with Gaucher disease and GBA heterozygotes. JAMA Neurol.

[7] Bultron G, Kacena K, Pearson D, Boxer M, Yang R, Sathe S (2010). The risk of Parkinson's disease in type 1 Gaucher disease. J Inherit Metab Dis.

[8] Clark LN, Kartsaklis LA, Wolf Gilbert R, Dorado B, Ross BM, Kisselev S (2009). Association of glucocerebrosidase mutations with dementia with Lewy bodies. Arch Neurol.

[9] Goker-Alpan O, Stubblefield BK, Giasson BI, Sidransky E (2010). Glucocerebrosidase is present in α-synuclein inclusions in Lewy body disorders. Acta Neuropathol.

[10] Neumann J, Bras J, Deas E, O'Sullivan SS, Parkkinen L, Lachmann RH (2009). Glucocerebrosidase mutations in clinical and pathologically proven Parkinson's disease. Brain.

[11] Tayebi N, Walker J, Stubblefield B, Orvisky E, LaMarca ME, Wong K (2003). Gaucher disease with parkinsonian manifestations: does glucocerebrosidase deficiency contribute to a vulnerability to parkinsonism?. Mol Genet Metab.

[12] Wong K, Sidransky E, Verma A, Mixon T, Sandberg GD, Wakefield LK (2004). Neuropathology provides clues to the pathophysiology of Gaucher disease. Mol Genet Metab.

[13] Du TT, Wang L, Duan CL, Lu LL, Zhang JL, Gao G (2015). GBA deficiency promotes SNCA/α-synuclein accumulation through autophagic inhibition by inactivated PPP2A. Autophagy.

[14] Fernandes HJ, Hartfield EM, Christian HC, Emmanoulidou E, Zheng Y, Booth H (2016). ER stress and autophagic perturbations lead to elevated extracellular α-synuclein in GBA-N370S Parkinson's iPSC-derived dopamine neurons. Stem Cell Rep.

[15] Ikuno M, Yamakado H, Akiyama H, Parajuli LK, Taguchi K, Hara J (2019). GBA haploinsufficiency accelerates alpha-synuclein pathology with altered lipid metabolism in a prodromal model of Parkinson's disease. Hum Mol Genet.

[16] Magalhaes J, Gegg ME, Migdalska-Richards A, Doherty MK, Whitfield PD, Schapira AH (2016). Autophagic lysosome reformation dysfunction in glucocerebrosidase deficient cells: relevance to Parkinson disease. Hum Mol Genet.

[17] Mazzulli JR, Xu YH, Sun Y, Knight AL, McLean PJ, Caldwell GA (2011). Gaucher disease glucocerebrosidase and α-synuclein form a bidirectional pathogenic loop in synucleinopathies. Cell.

[18] Schöndorf DC, Aureli M, McAllister FE, Hindley CJ, Mayer F, Schmid B (2014). iPSC-derived neurons from GBA1-associated Parkinson's disease patients show autophagic defects and impaired calcium homeostasis. Nat Commun.

[19] Sun Y, Grabowski GA (2010). Impaired autophagosomes and lysosomes in neuronopathic Gaucher disease. Autophagy.

[20] Taguchi YV, Liu J, Ruan J, Pacheco J, Zhang X, Abbasi J (2017). Glucosylsphingosine promotes α-synuclein pathology in mutant GBA-associated Parkinson's disease. J Neurosci.

[21] Kobayashi T, Suzuki K (1981). The glycosylceramidase in the murine intestine. Purification and substrate specificity. J Biol Chem.

[22] Citterio A, Arnoldi A, Panzeri E, D'Angelo MG, Filosto M, Dilena R (2014). Mutations in CYP2U1, DDHD2 and GBA2 genes are rare causes of complicated forms of hereditary spastic paraparesis. J Neurol.

[23] Hammer MB, Eleuch-Fayache G, Schottlaender LV, Nehdi H, Gibbs JR, Arepalli SK (2013). Mutations in GBA2 cause autosomal-recessive cerebellar ataxia with spasticity. Am J Hum Genet.

[24] Haugarvoll K, Johansson S, Rodriguez CE, Boman H, Haukanes BI, Bruland O (2017). GBA2 mutations cause a Marinesco-Sjögren-like syndrome: genetic and biochemical studies. PLoS ONE.

[25] Martin E, Schüle R, Smets K, Rastetter A, Boukhris A, Loureiro JL (2013). Loss of function of glucocerebrosidase GBA2 is responsible for motor neuron defects in hereditary spastic paraplegia. Am J Hum Genet.

[26] Sultana S, Reichbauer J, Schüle R, Mochel F, Synofzik M, van der Spoel AC (2015). Lack of enzyme activity in GBA2 mutants associated with hereditary spastic paraplegia/cerebellar ataxia (SPG46). Biochem Biophys Res Commun.

[27] Yildiz Y, Matern H, Thompson B, Allegood JC, Warren RL, Ramirez DM (2006). Mutation of beta-glucosidase 2 causes glycolipid storage disease and impaired male fertility. J Clin Invest.

[28] Yildiz Y, Hoffmann P, Vom Dahl S, Breiden B, Sandhoff R, Niederau C (2013). Functional and genetic characterization of the non-lysosomal glucosylceramidase 2 as a modifier for Gaucher disease. Orphanet J Rare Dis.

[29] Woeste MA, Stern S, Raju DN, Grahn E, Dittmann D, Gutbrod K (2019). Species-specific differences in nonlysosomal glucosylceramidase GBA2 function underlie locomotor dysfunction arising from loss-of-function mutations. J Biol Chem.

[30] Mistry PK, Liu J, Sun L, Chuang WL, Yuen T, Yang R (2014). Glucocerebrosidase 2 gene deletion rescues type 1 Gaucher disease. Proc Natl Acad Sci USA.

[31] Marques AR, Aten J, Ottenhoff R, van Roomen CP, Herrera Moro D, Claessen N (2015). Reducing GBA2 activity ameliorates neuropathology in Niemann-pick type C mice. PLoS ONE.

[32] Tybulewicz VL, Tremblay ML, LaMarca ME, Willemsen R, Stubblefield BK, Winfield S (1992). Animal model of Gaucher's disease from targeted disruption of the mouse glucocerebrosidase gene. Nature.

[33] Uemura N, Koike M, Ansai S, Kinoshita M, Ishikawa-Fujiwara T, Matsui H (2015). Viable neuronopathic Gaucher disease model in Medaka (*Oryzias latipes*) displays axonal accumulation of alpha-synuclein. PLoS Genet.

[34] Ansai S, Kinoshita M (2014). Targeted mutagenesis using CRISPR/Cas system in medaka. Biol Open.

[35] Akiyama H, Ide M, Nagatsuka Y, Sayano T, Nakanishi E, Uemura N (2020). Glucocerebrosidases catalyze a transgalactosylation reaction that yields a newly-identified brain sterol metabolite, galactosylated cholesterol. J Biol Chem.

[36] Overkleeft HS, Renkema GH, Neele J, Vianello P, Hung IO, Strijland A (1998). Generation of specific deoxynojirimycin-type inhibitors of the non-lysosomal glucosylceramidase. J Biol Chem.

[37] Ridley CM, Thur KE, Shanahan J, Thillaiappan NB, Shen A, Uhl K (2013). β-Glucosidase 2 (GBA2) activity and imino sugar pharmacology. J Biol Chem.

[38] Lee BR, Kamitani T (2011). Improved immunodetection of endogenous α-synuclein. PLoS ONE.

[39] Akiyama H, Nakajima K, Itoh Y, Sayano T, Ohashi Y, Yamaguchi Y (2016). Aglycon diversity of brain sterylglucosides: structure determination of cholesteryl- and sitosterylglucoside. J Lipid Res.

[40] Nakajima K, Akiyama H, Tanaka K, Kohyama-Koganeya A, Greimel P, Hirabayashi Y (2016). Separation and analysis of mono-glucosylated lipids in brain and skin by hydrophilic interaction chromatography based on carbohydrate and lipid moiety. J Chromatogr B.

[41] Aureli M, Bassi R, Loberto N, Regis S, Prinetti A, Chigorno V (2012). Cell surface associated glycohydrolases in normal and Gaucher disease fibroblasts. J Inherit Metab Dis.

[42] Burke DG, Rahim AA, Waddington SN, Karlsson S, Enquist I, Bhatia K (2013). Increased glucocerebrosidase (GBA) 2 activity in GBA1 deficient mice brains and in Gaucher leucocytes. J Inherit Metab Dis.

[43] Körschen HG, Yildiz Y, Raju DN, Schonauer S, Bönigk W, Jansen V (2013). The non-lysosomal β-glucosidase GBA2 is a non-integral membrane-associated protein at the endoplasmic reticulum (ER) and Golgi. J Biol Chem.

[44] Peri F, Nüsslein-Volhard C (2008). Live imaging of neuronal degradation by microglia reveals a role for v0-ATPase a1 in phagosomal fusion in vivo. Cell.

[45] Svennerholm L, Vanier MT, Månsson JE (1980). Krabbe disease: a galactosylsphingosine (psychosine) lipidosis. J Lipid Res.

[46] Zunke F, Moise AC, Belur NR, Gelyana E, Stojkovska I, Dzaferbegovic H (2018). Reversible conformational conversion of α-synuclein into toxic assemblies by glucosylceramide. Neuron.

[47] Klionsky DJ, Abdalla FC, Abeliovich H, Abraham RT, Acevedo-Arozena A, Adeli K (2012). Guidelines for the use and interpretation of assays for monitoring autophagy. Autophagy.

[48] Schapira AH (2015). Glucocerebrosidase and Parkinson disease: recent advances. Mol Cell Neurosci.

[49] Gan-Or Z, Amshalom I, Kilarski LL, Bar-Shira A, Gana-Weisz M, Mirelman A (2015). Differential effects of severe vs mild GBA mutations on Parkinson disease. Neurology.

[50] Sardi SP, Clarke J, Viel C, Chan M, Tamsett TJ, Treleaven CM (2013). Augmenting CNS glucocerebrosidase activity as a therapeutic strategy for parkinsonism and other Gaucher-related synucleinopathies. Proc Natl Acad Sci USA.

[51] Migdalska-Richards A, Daly L, Bezard E, Schapira AH (2016). Ambroxol effects in glucocerebrosidase and α-synuclein transgenic mice. Ann Neurol.

[52] Sardi SP, Viel C, Clarke J, Treleaven CM, Richards AM, Park H (2017). Glucosylceramide synthase inhibition alleviates aberrations in synucleinopathy models. Proc Natl Acad Sci USA.

[53] Dekker N, Voorn-Brouwer T, Verhoek M, Wennekes T, Narayan RS, Speijer D (2011). The cytosolic β-glucosidase GBA3 does not influence type 1 Gaucher disease manifestation. Blood Cells Mol Dis.

[54] Kim S, Yun SP, Lee S, Umanah GE, Bandaru VVR, Yin X (2018). GBA1 deficiency negatively affects physiological α-synuclein tetramers and related multimers. Proc Natl Acad Sci USA.

[55] Burré J (2015). The synaptic function of α-synuclein. J Parkinson’s Dis.

[56] Dettmer U, Selkoe D, Bartels T (2016). New insights into cellular α-synuclein homeostasis in health and disease. Curr Opin Neurobiol.

[57] Abdelkarim H, Marshall MS, Scesa G, Smith RA, Rue E, Marshall J (2018). alpha-Synuclein interacts directly but reversibly with psychosine: implications for alpha-synucleinopathies. Sci Rep.

[58] Smith BR, Santos MB, Marshall MS, Cantuti-Castelvetri L, Lopez-Rosas A, Li G (2014). Neuronal inclusions of α-synuclein contribute to the pathogenesis of Krabbe disease. J Pathol.

[59] Hallett PJ, Huebecker M, Brekk OR, Moloney EB, Rocha EM, Priestman DA (2018). Glycosphingolipid levels and glucocerebrosidase activity are altered in normal aging of the mouse brain. Neurobiol Aging.

