# Supplementary figures and images for: Impact of Gba2 on neuronopathic Gaucher’s disease and α-synuclein accumulation in medaka (Oryzias latipes)
Source: Mol Brain. 2021 May 10;14:80. doi: 10.1186/s13041-021-00790-x (PMC8111776; doi:10.1186/s13041-021-00790-x)

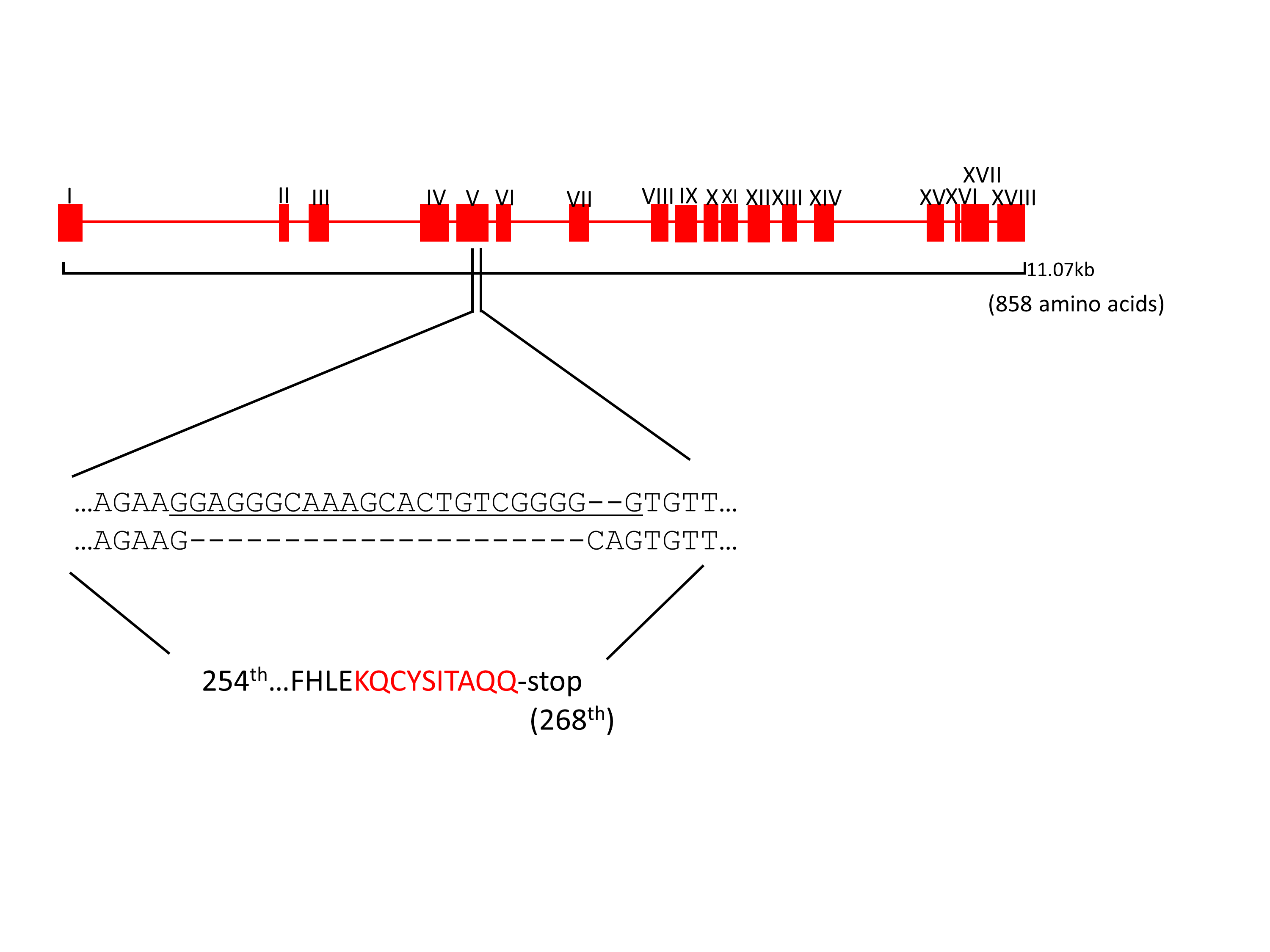

Supplement: Supplementary file 1 — Additional file 1: Figure S1. Genome structure of medaka GBA2 and mutations induced by CRISPR/Cas9. The red boxes represent exons, and the Greek numbers indicate the exon number. Medaka GBA2 consists of 2577 bases and 858 amino acids. The deletion of 21 bases and insertion of 2 bases in exon 5 of gba2 led to a nonsense mutation. The underlined part represents the used crRNA sequence to generate the gba2 KO medaka in this study. [file 13041_2021_790_MOESM1_ESM.tif]

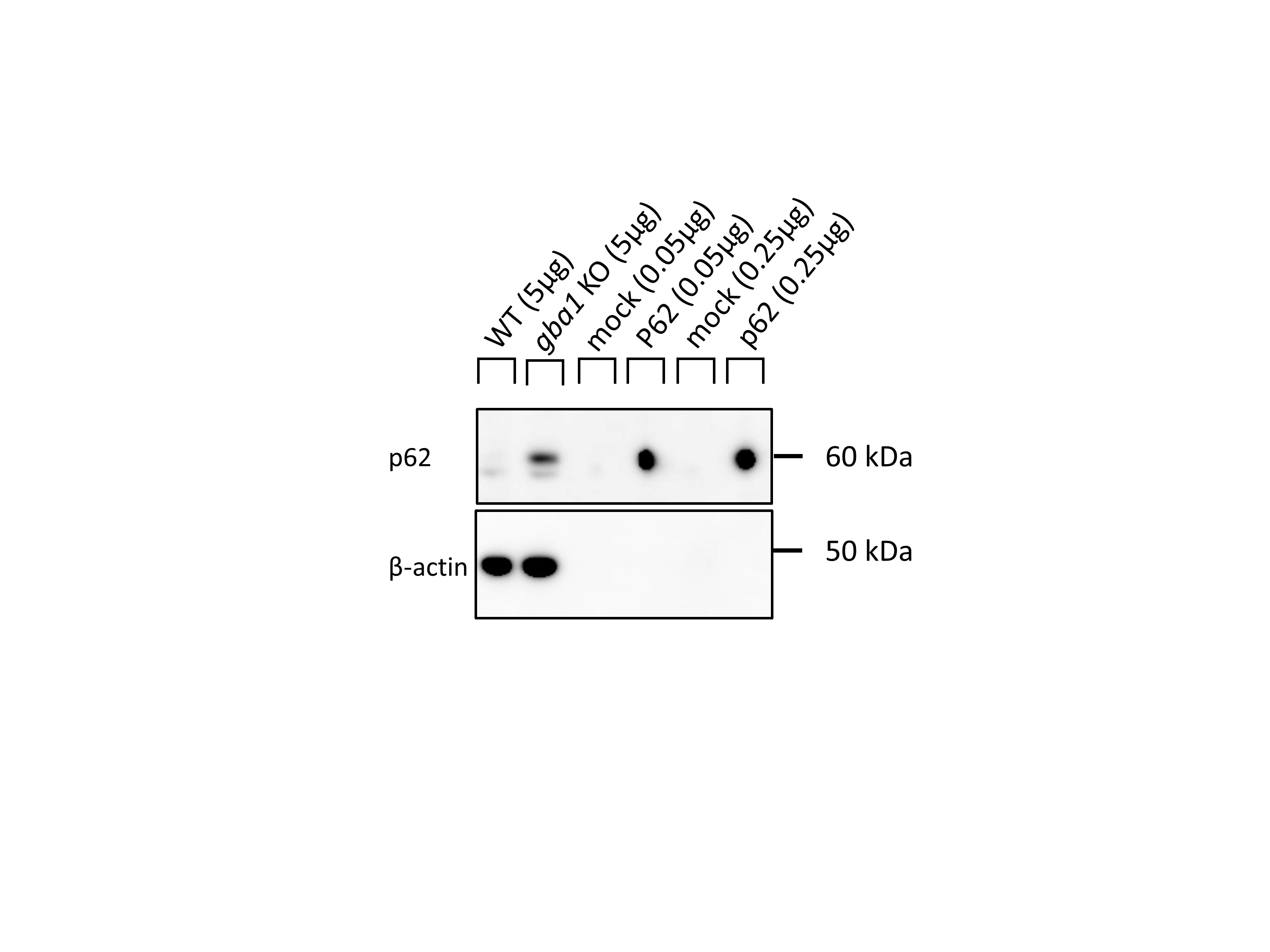

Supplement: Supplementary file 2 — Additional file 2: Figure S2. Confirmation of the cross-species reactivity of the p62 antibody. A strong signal was detected at the molecular weight close to that of medaka p62 in the lanes of medaka p62-overexpressing HEK293T cells and gba1 KO medaka brains, but not in the lane of mock transfected cells. [file 13041_2021_790_MOESM2_ESM.tif]

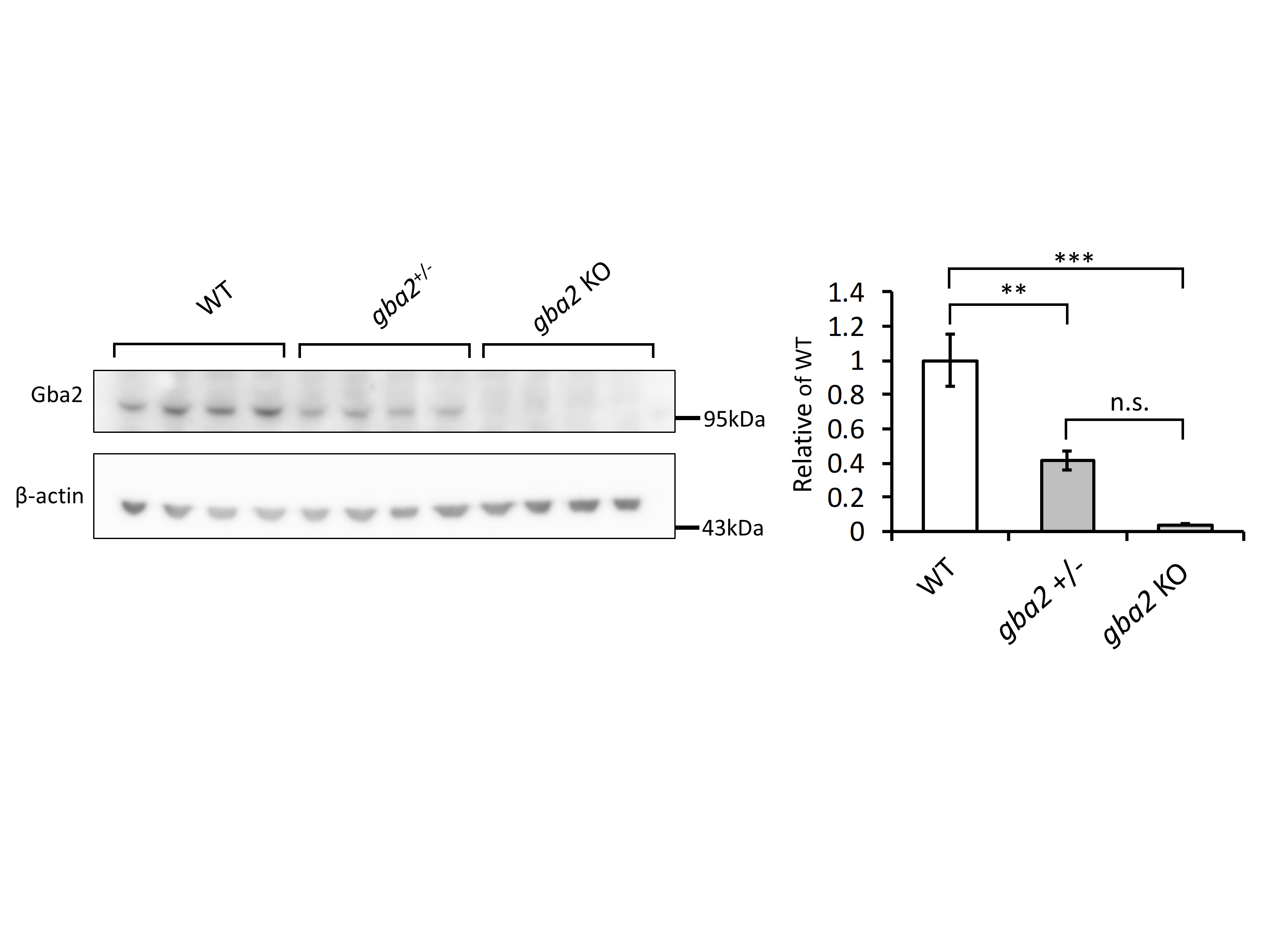

Supplement: Supplementary file 5 — Additional file 5: Figure S3. Gba2 protein expression in gba2 mutant medaka. Immunoblot analysis showed almost no expression of Gba2 protein in the brains of gba2 KO medaka at 3 mpf. gba2+/− medaka showed ~ 42% of Gba2 expression level compared with WT. (n = 4 for each genotype). n.s.: not significant, **p < 0.01 and ***p < 0.001 (a one-way ANOVA with Tukey's multiple comparison test). The bars indicate the mean ± SEM. [file 13041_2021_790_MOESM5_ESM.tif]

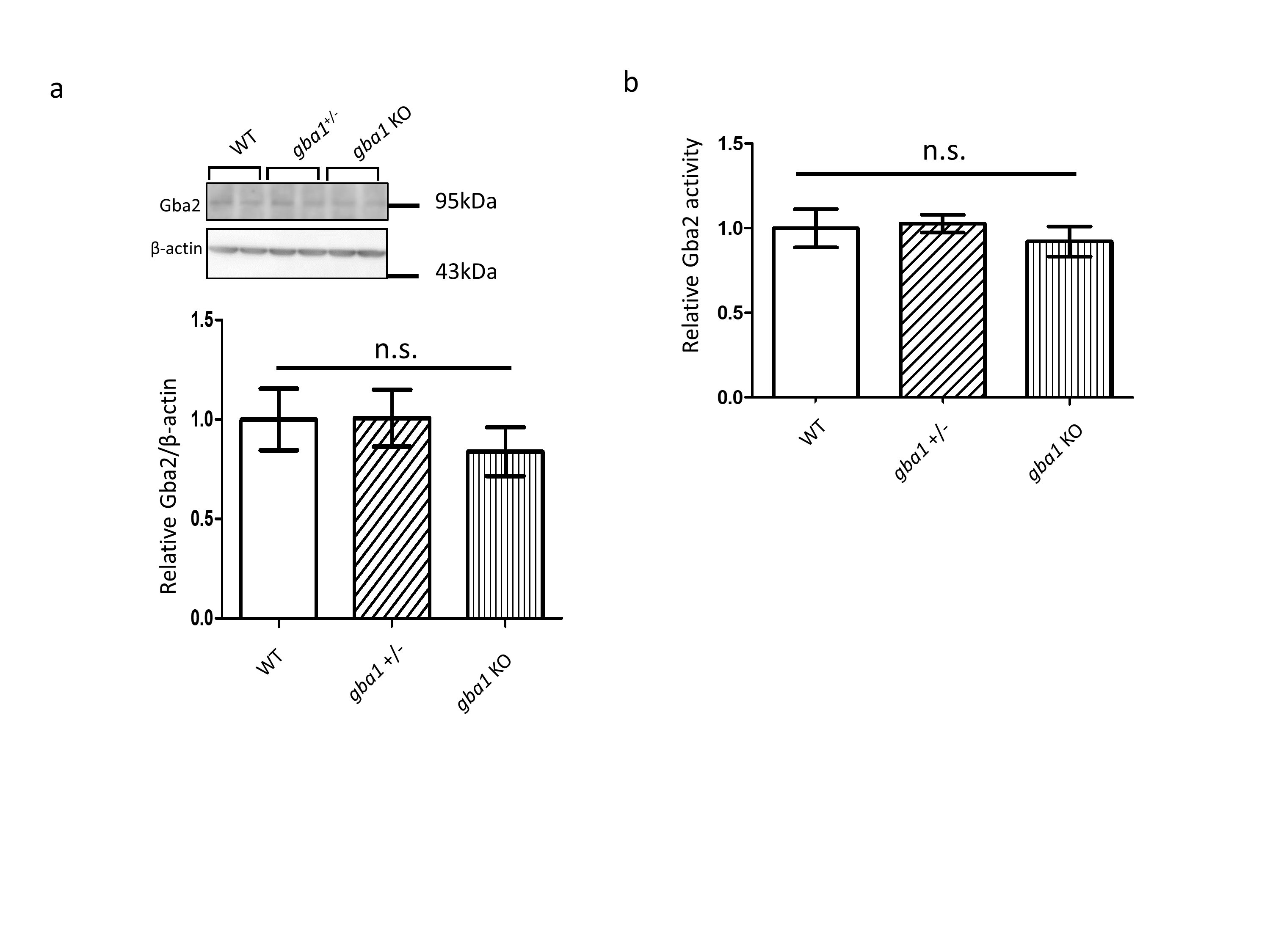

Supplement: Supplementary file 6 — Additional file 6: Figure S4. Gba2 protein expression and enzymatic activity in gba1 mutant medaka. (a) Immunoblot analysis of Gba2 in the brains of gba1 mutant medaka at 3 mpf. The Gba2 protein expression did not significantly differ among genotypes (n = 4 for each genotype). (b) The Gba2 enzymatic activity of gba1 mutant medaka at 3 mpf. The Gba2 activity did not significantly differ among genotypes (n = 8 for each group). A one-way ANOVA with Tukey's multiple comparison test was performed. n.s.: not significant. The bars indicate the mean ± SEM. [file 13041_2021_790_MOESM6_ESM.tif]

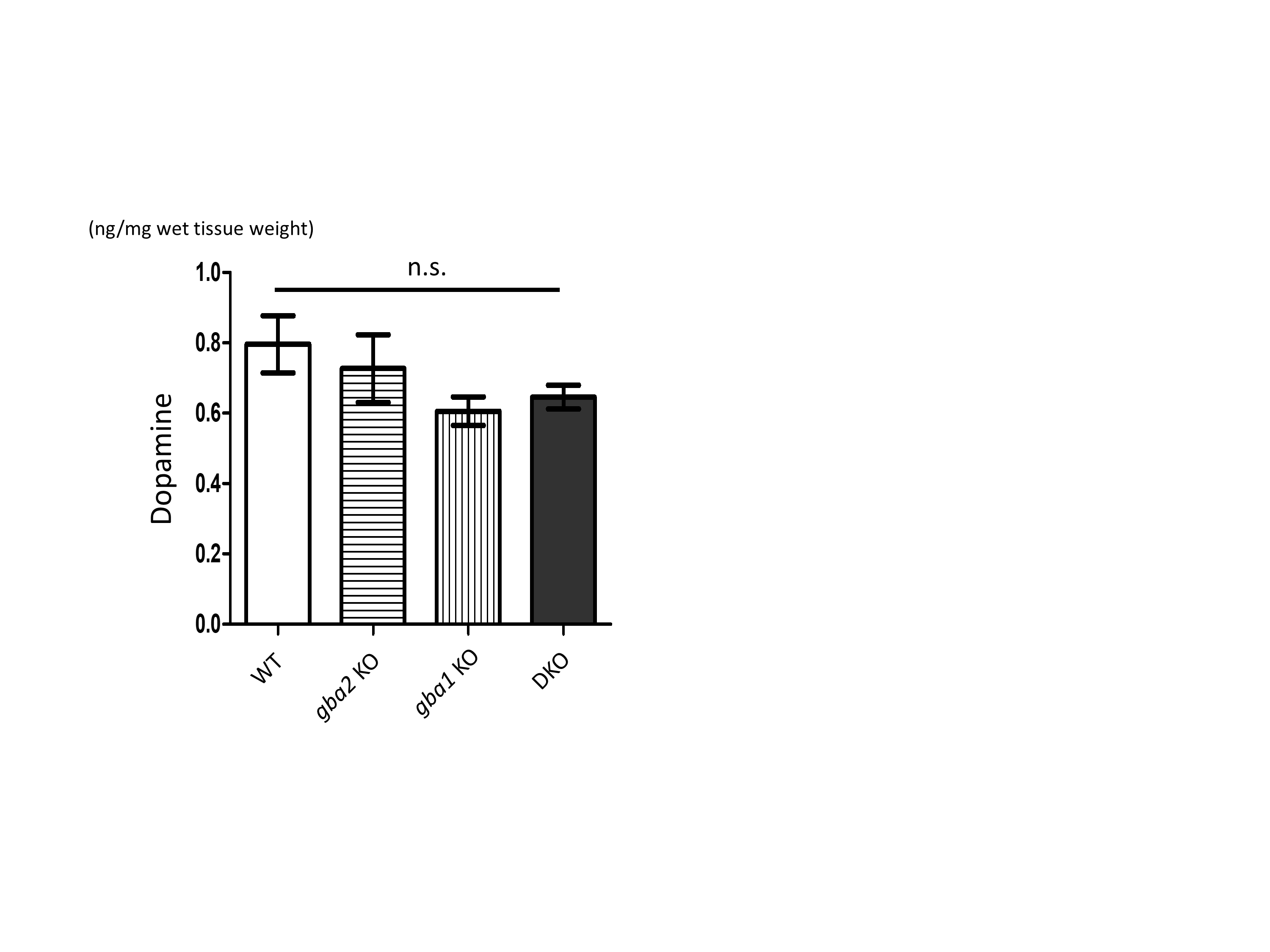

Supplement: Supplementary file 11 — Additional file 11: Figure S5. Dopamine measurement of the brains by high performance liquid chromatography. Dopamine measurement of the brains was conducted at 3mpf. The data showed trend towards decrease in the amount of dopamine in the brains of gba1 KO and gba1/gba2 DKO medaka albeit not reaching statistical significance (n = 4 for each genotype). A one-way ANOVA with Tukey's multiple comparison test was performed. n.s.: not significant. [file 13041_2021_790_MOESM11_ESM.tif]

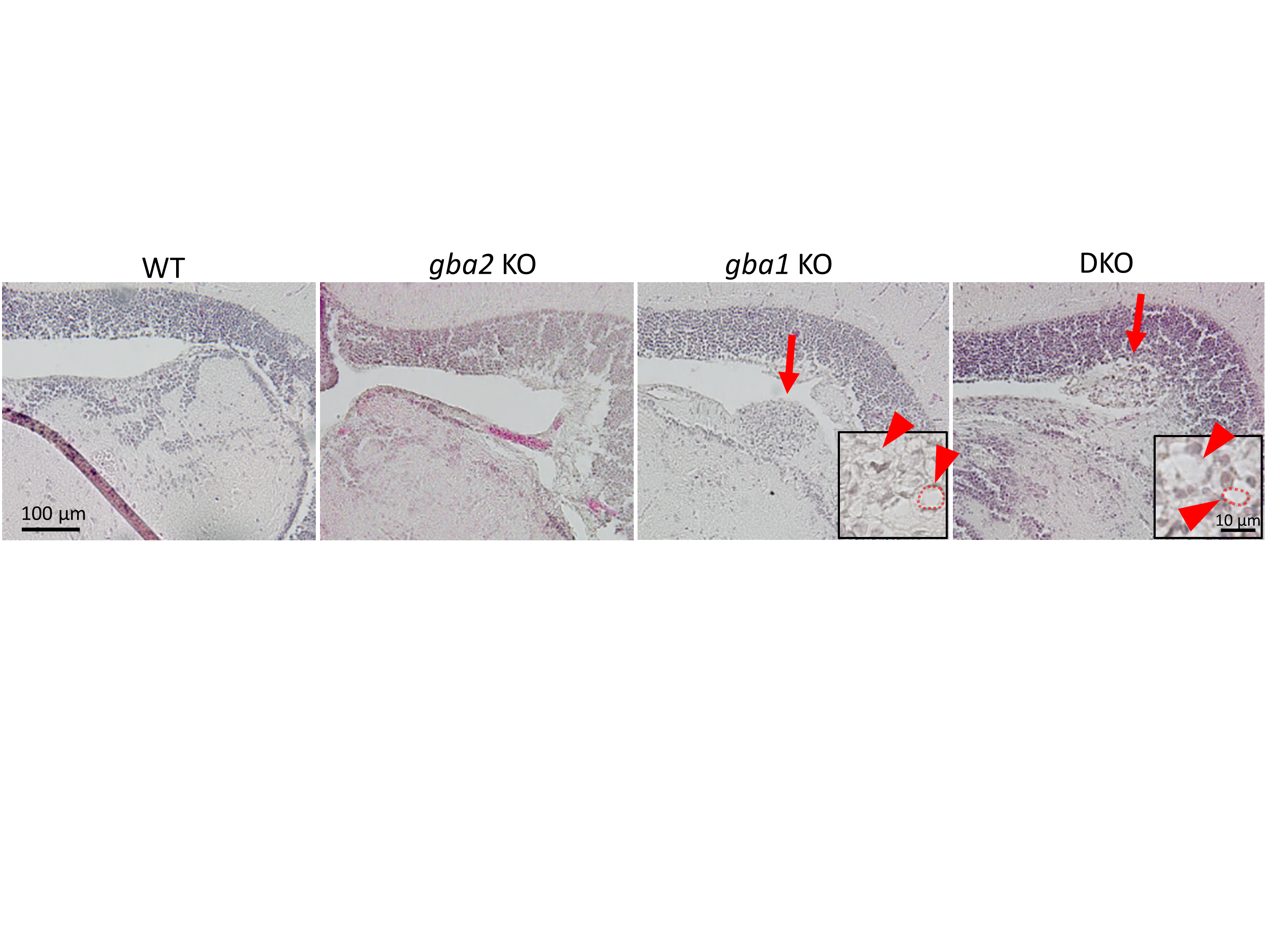

Supplement: Supplementary file 12 — Additional file 12: Figure S6. Gaucher cell-like cells appeared in gba1/gba2 DKO as well as gba1 KO medaka brains. Hematoxylin and eosin staining showed abnormal cells in the periventricular gray zone of the optic tectum in gba1/gba2 DKO and gba1 KO medaka but not in the WT or gba2 KO medaka (arrows). Enlarged images of Gaucher cell-like cells containing large vacuoles are shown in the insets (arrowheads). The representative Gaucher cell-like cells are circled in dot red line. [file 13041_2021_790_MOESM12_ESM.tif]

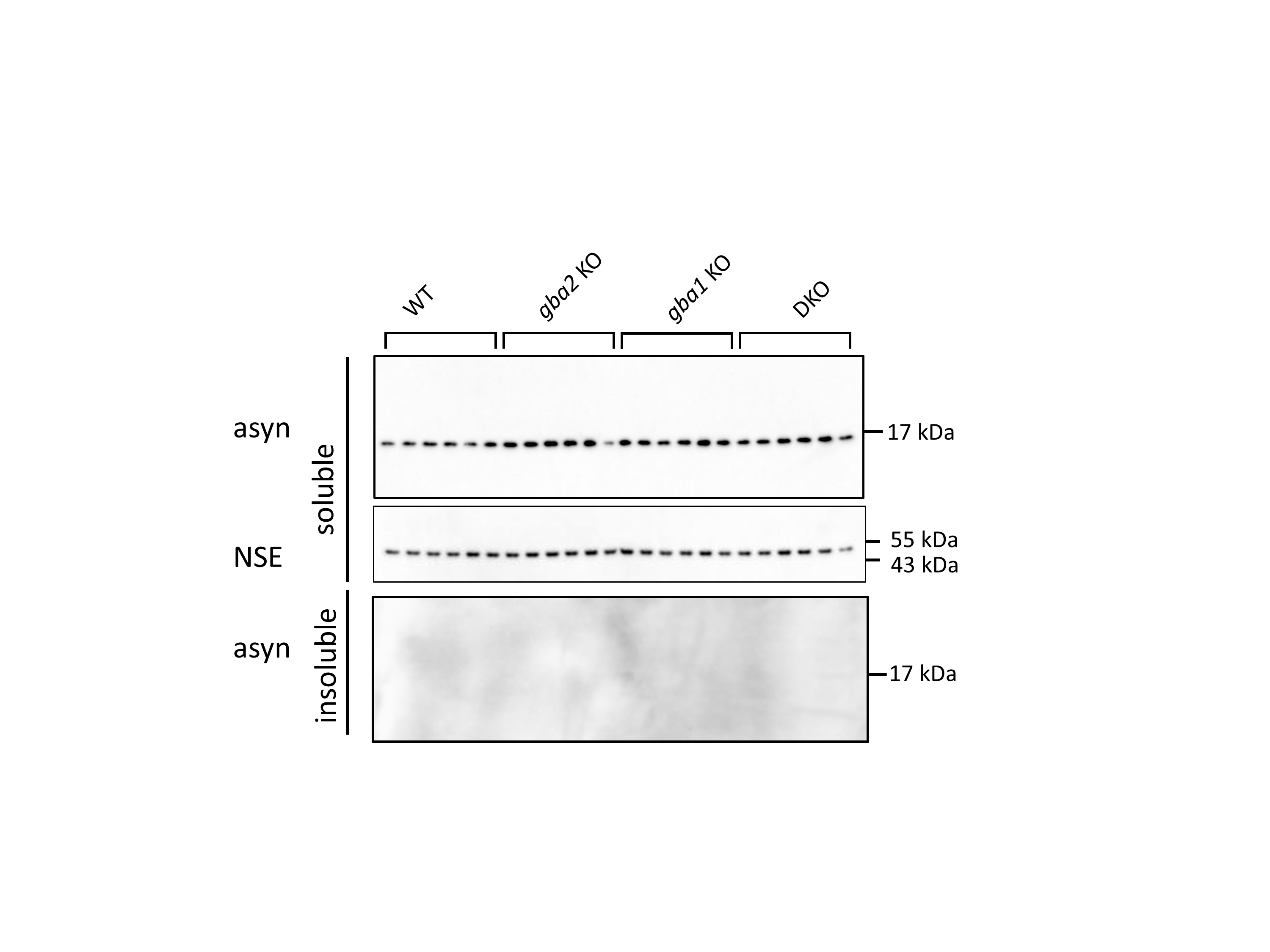

Supplement: Supplementary file 14 — Additional file 14: Figure S7. Expression of asyn in Triton X-insoluble, SDS-soluble fractions. Expression of asyn was not detected in Triton X-insoluble, SDS-soluble fractions (shown “insoluble” in the figure). The immunoblot data of the Triton X-soluble (shown “soluble” in the figure) is the same as Fig. 4a. [file 13041_2021_790_MOESM14_ESM.tif]

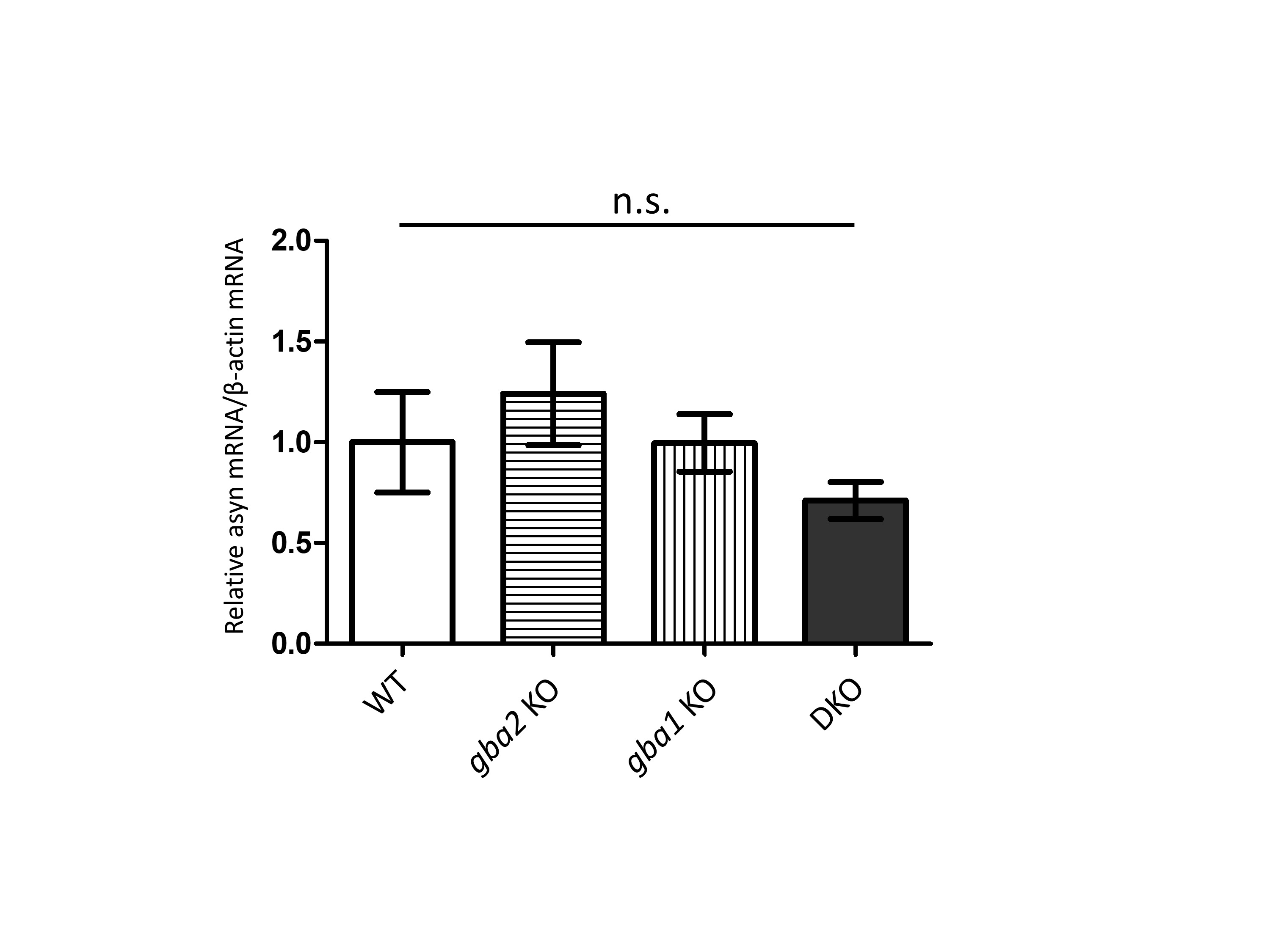

Supplement: Supplementary file 15 — Additional file 15: Figure S8. gba2 mRNA expression in brains measured by qRT-PCR. Measurement of asyn mRNA expression in the brains was conducted at 3 mpf. (n = 4 for each genotypes). A one-way ANOVA with Tukey's multiple comparison test was performed. n.s.: not significant. [file 13041_2021_790_MOESM15_ESM.tif]
