# Supplementary material for: Impact of Gba2 on neuronopathic Gaucher’s disease and α-synuclein accumulation in medaka (Oryzias latipes)
Source: Mol Brain. 2021 May 10;14:80. doi: 10.1186/s13041-021-00790-x (PMC8111776; doi:10.1186/s13041-021-00790-x)
Supplement: Supplementary file 3 — Additional file 3: Table S1. Analytical conditions used for the analysis by MRM methods. [file 13041_2021_790_MOESM3_ESM.pdf]

Analyte	Precursor ion (Q1) $[M + H]^+$	Product ion (Q3) long-chain base-related ions	Collision energy (CE) (eV)
d17:1-Sphingosine (C17 base)	286.4	268.3	15
Sphingosine (C18 base)	300.1	282.5	15
Ceramide (d18:1-C12:0)	482.6	264.4	25.5
Ceramide (d18:1-C16:0)	538.7	264.4	30.5
Ceramide (d18:1-C18:0)	566.5	264.4	33.0
Ceramide (d18:1-C24:1)	648.9	264.4	35.5
Ceramide (d18:1-C24:0)	650.9	264.4	35.5
GlcSph and GalSph	462.3	282.1	27
GlcSph-*d*5	467.3	287.1	27
GlcCer (d18:1-C12:0)	644.3	264.2	43
GlcCer and GalCer (d18:1-C14:0)	672.7	264.2	44.25
GlcCer and GalCer (d18:1-C16:0)	700.7	264.2	45.5
GlcCer and GalCer (d18:1-C18:1)	726.7	264.2	48
GlcCer and GalCer (d18:1-C18:0)	728.7	264.2	48
GlcCer and GalCer (d18:1-C20:1)	754.7	264.2	50.5

GlcCer and GalCer (d18:1-C20:0)	756.7	264.2	50.5
GlcCer and GalCer (d18:1-C22:1)	782.7	264.2	53
GlcCer and GalCer (d18:1-C22:0)	784.7	264.2	53
GlcCer and GalCer (d18:1-C23:1)	796.7	264.2	54.25
GlcCer and GalCer (d18:1-C23:0)	798.7	264.2	55
GlcCer and GalCer (d18:1-C24:1)	810.7	264.2	55.5
GlcCer and GalCer (d18:1-C24:0)	812.7	264.2	56.25
GlcCer and GalCer (d18:1-C26:1)	838.8	264.2	58
