# Supplementary material for: Impact of Gba2 on neuronopathic Gaucher’s disease and α-synuclein accumulation in medaka (Oryzias latipes)
Source: Mol Brain. 2021 May 10;14:80. doi: 10.1186/s13041-021-00790-x (PMC8111776; doi:10.1186/s13041-021-00790-x)
Supplement: Supplementary file 4 — Additional file 4: Table S2. Off-target candidates of the crRNA used to generate gba2 KO medaka in the present study. These candidates were identified with the Medaka pattern match tool (http://viewer.shigen.info/medakavw/crisprtool/). Three out of the 15 candidates were found in exons. [file 13041_2021_790_MOESM4_ESM.pdf]

Chromosome	Start(bp)	End(bp)	Code
1	31730386	31730408	Non oode
2	29443195	29443217	Exon(novel)
6	673883	673861	Intron(grm8b)
6	26002621	26002599	Intron(novel)
9	4460829	4460807	Non code
10	4953165	4953143	Non code
11	448194	448172	Non code
11	23336776	23336754	Non code
11	23610753	23610731	Exon(rpl30)
12	6993059	6993081	Non code
13	1036572	1036550	No code
15	15662890	15662912	Exon ppm1b
15	17109266	17109244	Non code
18	15330018	15329996	Non code
24	2973433	2973455	Non code
