# Supplementary material for: Impact of Gba2 on neuronopathic Gaucher’s disease and α-synuclein accumulation in medaka (Oryzias latipes)
Source: Mol Brain. 2021 May 10;14:80. doi: 10.1186/s13041-021-00790-x (PMC8111776; doi:10.1186/s13041-021-00790-x)
Supplement: Supplementary file 13 — Additional file 13: Table S3. The sphingolipid profiles in each genotype. The amount of each sphingolipid in each genotype was compared with that in WT (n = 3 for each genotype). N.D.: not detected (below the detection threshold). n.s.: not significant, *p < 0.05, **p < 0.01, ***p < 0.001 and ****p < 0.0001 (a two-tailed unpaired Student’s t-test). [file 13041_2021_790_MOESM13_ESM.pdf]

Genotype		GlcCer ±SEM (pmol/mg wet weight)	Statistical significance	GalCer ±SEM (pmol/mg wet weight)	Statistical significance	GlcSph ±SEM (pmol/mg wet weight)	Statistical significance
gba1	*gba2*						
+/+	+/+	2.59 ± 0.25		337.31 ±24.32		N.D.	
+/+	+/-	3.77 ± 0.49	n.s.	298.44 ±37.39	n.s.	N.D	n.s.
+/+	-/-	82.81 ±1.04	****	296.30 ±25.77	n.s.	N.D.	n.s.
+/-	+/+	2.11 ±0.22	n.s.	321.41 ±34.91	n.s.	N.D.	n.s.
+/-	+/-	17.37 ±13.33	n.s.	290.32 ±5.24	n.s.	0.06 ±0.06	*
+/-	-/-	81.10 ±8.21	***	294.10 ±25.86	n.s.	0.41 ±0.09	**
-/-	+/+	266.54 ±26.84	***	123.08 ±9.99	**	7.06 ±0.45	****
-/-	+/-	277.78 ±34.41	**	124.12 ±19.77	**	7.90 ±0.7	**
-/-	-/-	526.31 ±70.8	**	138.36 ±14.2	**	13.10 ±0.88	***

Genotype		GalSph ±SEM (pmol/mg wet weight)	Statistical significance	Ceramide ±SEM (pmol/mg wet weight)	Statistical significance	Sphingosine ±SEM (pmol/mg wet weight)	Statistical significance
gba1	*gba2*						
+/+	+/+	N.D.		104947.60 ±13291.13		2.20 ±0.06	
+/+	+/-	N.D.	n.s.	113315.48 ±10183.51	n.s.	2.59 ±0.042	n.s.
+/+	-/-	0.49 ±0.09	**	85185.32 ±6512.23	n.s.	2.09 ±0.06	n.s.
+/-	+/+	0.50 ±0.15	*	99116.09 ±9634.56	n.s.	2.22 ±0.04	n.s.
+/-	+/-	0.50 ±0.12	*	83621.49 ±14867.63	n.s.	2.32 ±0.24	n.s.
+/-	-/-	0.56 ±0.17	*	77961.84 ±12308.50	n.s.	2.22 ±0.19	n.s.
-/-	+/+	1.69 ±0.09	***	62110.95 ±6655.21	*	3.22 ±0.31	*
-/-	+/-	1.70 ±0.24	**	59826.72 ±8820.32	*	3.13 ±0.38	n.s.
-/-	-/-	2.39 ±0.76	*	58860.60 ±9106.26	*	4.05 ±0.43	*
